# Matrix-biased excitatory and inhibitory inputs to the striatum involving external segment of the globus pallidus

**DOI:** 10.3389/fncel.2025.1706469

**Published:** 2025-11-19

**Authors:** Fuyuki Karube, Kenta Kobayashi, Fumino Fujiyama

**Affiliations:** 1 Laboratory Cytology and Histology, Graduate School of Medicine, Hokkaido University, Sapporo, Japan; 2 Section of Viral Vector Development, National Institute for Physiological Sciences, Okazaki, Japan

**Keywords:** external segment of globus pallidus (GPe), striatum, cholinergic interneurons, matrix, striosome

## Abstract

**Introduction:**

The external segment of the globus pallidus (GPe) is traditionally viewed as a relay nucleus within the indirect basal ganglia pathway. However, a subpopulation of GPe neurons projects directly to the striatum, raising questions about their compartmental and cell-type-specific targeting.

**Methods:**

To address this issue, we employed neural tracing and *ex vivo* whole-cell patch-clamp recordings with optogenetics using adeno-associated viral vectors in rats. Anatomical observations and intersectional labeling techniques were applied to examine spatial relationships of projections among the striatum, GPe, and ventral thalamus.

**Results:**

GPe axons exhibited a strong bias toward the matrix compartment of the striatum. This biased projection originated from both subthalamic nucleus-targeting and striatum-targeting GPe neurons. In contrast, striatal projections to the GPe arose from both matrix and striosome compartments. Optogenetic stimulation of GPe axons elicited inhibitory postsynaptic currents in medium spiny neurons (MSNs) and cholinergic interneurons (CINs) in the matrix compartment. Cesium-based recordings indicated distal synaptic contacts in MSNs. Anatomical data also revealed proximal appositions of GPe axons to CIN somata and dendrites. Excitatory inputs from motor cortical areas and ventral thalamic nuclei also preferentially targeted the matrix. Furthermore, optogenetic stimulation of ventral thalamic axons elicited excitatory postsynaptic currents in GPe neurons. Intersectional labeling revealed substantial overlap between striatal neurons and axons of GPe neurons, both of which were innervated by the same population of ventral thalamic neurons.

**Discussion:**

These findings suggest that convergent cortical and thalamic excitation of both the striatum and GPe may induce feedforward inhibition within the striatal matrix, particularly onto CINs. This mechanism may contribute to the fine-tuning of striatal output in motor-related basal ganglia circuits.

## 1 Introduction

The basal ganglia comprise a group of subcortical nuclei that are essential for selecting and initiating adaptive behaviors, such as goal-directed movement and decision-making. These nuclei include the striatum, external segment of the globus pallidus (GPe), subthalamic nucleus (STN), internal segment of the globus pallidus internal segment (GPi, the counterpart of the entopeduncular nucleus, EP, in rodents), and substantia nigra (SN). According to the widely accepted model of the basal ganglia (Albin et al., 1989; Alexander and Crutcher, 1990; Redgrave et al., 2011; Arber and Costa, 2022), the striatum and STN receive cortical inputs and function as input nuclei of the basal ganglia. Subsequently, two distinct types of GABAergic projection neurons form the direct and indirect pathways: direct pathway medium spiny neurons (dMSNs) innervate the GPi/EP and SN, while indirect pathway medium spiny neurons (iMSNs) project to the GPe. Since the GPe ultimately targets GPi/EP and SNr, the direct and indirect pathways exert opposing effects. Maintaining an appropriate balance between these pathways is crucial for coordinated motor behavior (Friend and Kravitz, 2014; Shipp, 2017). Indeed, pathological impairments of the basal ganglia, such as those seen in Parkinson’s disease (PD), disrupt this balance and lead to impaired voluntary movement.

Recent findings have offered a new perspective on the functions of the GPe, which has traditionally been considered merely a relay nucleus of the indirect pathway (for reviews, Hegeman et al., 2016; Courtney and Chan, 2023; Fang and Creed, 2024; Giossi et al., 2024). A key advance is the identification of two distinct types of projection neurons in the GPe: prototypic neurons and arkypallidal neurons (Mallet et al., 2012; Abdi et al., 2015; Hernandez et al., 2015; Fujiyama et al., 2016). Prototypic neurons constitute the major population and project primarily to the downstream nuclei of the basal ganglia, including the STN, GPi/EP, and SN. These neurons have long been recognized as a classical type of GPe neuron. In contrast, arkypallidal neurons project exclusively to the striatum, lacking axon collaterals to downstream nuclei. In addition, the molecular identities of these two types of GPe neurons are markedly distinct. Prototypic neurons predominantly express parvalbumin (PV) and/or Nkx2.1, whereas arkypallidal neurons exclusively express FoxP2. Expression of Npas1 is observed in both arkypallidal (∼60%) and non-arkypallidal neurons (∼40%) (Abdi et al., 2015; Hernandez et al., 2015; Cui et al., 2021a). Although not incorporated into traditional models, arkypallidal neurons account for approximately one-fourth to one-third of the total GPe population and send dense axonal projections to the striatum. Building on pioneering studies, their anatomical, physiological, and functional characteristics have begun to emerge (for reviews, Aristieta and Gittis, 2021; Courtney and Chan, 2023; Fang and Creed, 2024). Pallidostriatal axons target both striatal projection neurons and interneurons with cell type-specific selectivity (Mallet et al., 2012; Glajch et al., 2016; Klug et al., 2018). Ketzef and Silberberg (2021) revealed that prototypic neurons receive stronger inputs from iMSNs than from dMSNs, while arkypallidal neurons primarily receive dMSN inputs, based on *in vivo* whole-cell recordings in mice. Cui et al. (2021a) confirmed similar connectivity from the striatum to GPe neurons and reported changes in postsynaptic currents in PD model mice. Cortical innervation onto GPe neurons has been observed in mice (Abecassis et al., 2020) and rats (Karube et al., 2019), although the specificity of cell types remains controversial (Courtney and Chan, 2023). Cui et al. (2021b) further demonstrated the cellular diversity of GPe neurons in detail. In addition, their functional roles have been elucidated under both normal and pathological conditions (Vitek et al., 2012; Aristieta et al., 2021; Cui et al., 2021a,b; Courtney et al., 2023; Labouesse et al., 2023). Arkypallidal neurons have been shown to contribute specifically to the cancelation of prepared behavior in rats (Mallet et al., 2016). Similar trends have been reported in mice, where Npas1-positive GPe neurons suppress motor behavior (Glajch et al., 2016; Aristieta et al., 2021) and modulate various parameters of movement (Cui et al., 2021b; Labouesse et al., 2023). In parallel, computational studies have begun to incorporate emerging anatomical and physiological features of GPe neurons, suggesting their potential roles in action selection, action interruption, and pathological oscillations (Bogacz et al., 2016; Corbit et al., 2016; Suryanarayana et al., 2019; Gast et al., 2021; Goenner et al., 2021). Therefore, pallidostriatal innervations—long overlooked in canonical models of the basal ganglia—have attracted increasing attention, although their presence in primates has yet to be confirmed (but see Sato et al., 2000 for morphological observation). These recent insights into GPe cell types raise the question of how their projections interact with the compartmental architecture of the striatum, particularly between the striosome and matrix.

The striatum is subdivided into two compartments: the striosome and matrix, which differ in their molecular markers, connectivity, and functional properties (for review, Crittenden and Graybiel, 2011). The striosome forms a continuous but irregularly shaped structure, appearing as patchy or island-like regions in anatomical sections. Occupying approximately 10–20% of the total striatal volume, the striosome is embedded within the surrounding matrix, which spans a broader territory. This compartmental dichotomy is supported by diverse lines of evidence, including differential gene and protein expression, distinct afferent inputs, and divergent projection targets (Gerfen, 1984, 1989). For example, mu-opioid receptor (MOR) is expressed selectively in the striosome, whereas calbindin D-28k (CB) is strongly expressed in the matrix (Herkenham and Pert, 1981; Gerfen, 1984, 1985; Ito et al., 1992; Liu and Graybiel, 1992). Cortical innervation also differs: the striosome receives inputs primarily from prefrontal and limbic areas, while the matrix is innervated by motor and sensory areas (Crittenden and Graybiel, 2011). Nevertheless, subregions encompassing both striosome islands and adjacent matrix are innervated by related cortical areas (Ragsdale and Graybiel, 1990; Flaherty and Graybiel, 1995; Kincaid and Wilson, 1996). Thalamic innervation further highlights this contrast: the striosome receives sparse vesicular glutamate transporter 2 (VGluT2)-positive inputs, whereas the matrix is densely innervated by VGluT2-positive axon terminals (Fujiyama et al., 2006; Raju et al., 2006). Notably, the compartmental preference of thalamic projections depends on the origin of the thalamic nuclei—for example, intralaminar nuclei, including the parafascicular nucleus (PaF) preferentially target the matrix, while midline nuclei favor the striosome (Herkenham and Pert, 1981; Ragsdale and Graybiel, 1991; Sadikot et al., 1992; Unzai et al., 2017). Regarding cell type composition, dMSNs appear to be overrepresented in the striosome, whereas both dMSNs and iMSNs are present in approximately equal proportions within the matrix (Levesque and Parent, 2005; Fujiyama et al., 2011). Importantly, the dendritic arbors of MSNs are generally confined to the compartment in which their cell bodies reside (Kawaguchi et al., 1989; Walker et al., 1993), thereby limiting the integration of signals across compartment boundaries. In contrast, parvalbumin (PV)-positive interneurons possess dendrites and axons that extend beyond the compartment borders. Kubota and Kawaguchi (1993) reported that cholinergic, PV-, or nitric oxide synthase -positive interneurons are predominantly located in the matrix, with a smaller subset at the border between the striosome and matrix, and only a few within the striosome in rats. Thus, PV interneurons may facilitate cross-compartmental inhibition, potentially contributing to the integration of signals between striosome and matrix (Cowan et al., 1990). Similar morphological features have also been reported for other striatal interneuron types, including cholinergic interneurons (CINs) (Kawaguchi, 1992). The distribution of these interneurons exhibits slight interspecies differences (Jakab et al., 1996; Holt et al., 1997; Bernácer et al., 2007, 2012), which may reflect both chemical and structural heterogeneity in striatal organization. The dendrites of CINs extend across compartmental borders and their axons innervate the matrix, suggesting an ability for cross-compartmental integration (Miura et al., 2008).

The input-output relationship involving dopaminergic neurons also differ between the matrix and striosome compartments. Striosomal neurons preferentially project to the substantia nigra pars compacta (SNc) (Gerfen, 1984; Fujiyama et al., 2011; Crittenden et al., 2016; McGregor et al., 2019; but see also Smith et al., 2016). Individual SNc dopaminergic neurons innervate both compartments (Matsuda et al., 2009). However, dopamine may exert striatal compartment-specific effects depending on differences in cell types and receptor expression. Indeed, dopaminergic modulation in the matrix differs from that in the striosome (Prager et al., 2020), where neurons exhibit distinct activity related to reward and punishment (Yoshizawa et al., 2018). These distinctions suggest that dopaminergic innervation may differentially modulate excitatory inputs across striatal compartments, potentially leading to compartment-specific biases in excitation and inhibition. Particularly, whether such biases are counterbalanced by compartment-selective inhibitory innervations remains an open question.

To address this issue, we examined the distribution and compartmental preference of inhibitory projections to the striatum, focusing on GABAergic inputs from the GPe. Previous studies have shown that various striatal neurons—including dMSNs, iMSNs, GABAergic interneurons, and CINs—are innervated by GPe neurons, including both prototypic and arkypallidal types (Bevan et al., 1998; Mallet et al., 2012; Hernandez et al., 2015). However, the distribution of these striatal cell types differs between the striosome and matrix compartments (for review, Crittenden and Graybiel, 2011), suggesting that compartment-specific differences in GPe-mediated inhibition may arise. *In vivo* recordings have revealed that arkypallidal neurons are spontaneously inactive and fire in-phase with cortical active states (Mallet et al., 2012; Abdi et al., 2015; Dodson et al., 2015; Ketzef and Silberberg, 2021), implying that direct or indirect excitation will be required to recruit arkypallidal neurons. Furthermore, cortical axons that innervate the striatum have also been shown to activate GPe neurons (Karube et al., 2019; Abecassis et al., 2020), suggesting that the striatum and GPe may share common cortical inputs. If this is the case, GPe projections to the striatum could function as a feedforward inhibitory mechanism, enabling the precise temporal control of striatal phasic activity. Moreover, given the compartment-selective nature of thalamic inputs, the thalamus may exert analogous modulatory effects. In addition, anatomical observations by Mallet et al. (2012) emphasize the presence of proximal GPe inputs onto striatal interneurons, including CINs. Combined with the predominance of interneurons in the matrix in the rat striatum (Kubota and Kawaguchi, 1993), these findings suggest that GPe inputs may differ in their targeting of MSNs versus interneurons. Among striatal interneurons, we focus on CINs in this study, as they are a major interneuron type within the matrix and receive substantial input from GPe neurons. These cells play a key role in modulating striatal activity underlying motor and cognitive flexibility, making their connectivity particularly important for understanding basal ganglia function. Therefore, in this study, we aimed to clarify the relationship between inhibitory projections from the GPe and the compartmental organization of the striatum, as well as to explore how cortical and thalamic innervation interact with this inhibitory circuitry.

## 2 Materials and methods

### 2.1 Animals

All experimental procedures involving animals were approved by the Animal Care and Use Committee of Hokkaido University (Approval No. 20-0106). Procedures involving adeno-associated viral (AAV) vectors were additionally approved by the Safety Committee on Genetic Recombination Experiments Hokkaido University (Approval No. 2020-019). All experiments were conducted in accordance with the National Institutes of Health Guide for the Care and Use of Laboratory Animals (NIH Publications No. 80–23) revised in 1996, the UK Animals (Scientific Procedures) Act 1986 and associated guidelines, and the European Communities Council Directive of November 24, 1986 (86/609/EEC). Every effort was made to minimize the number of animals used and to reduce their suffering. In total, sixty male Wistar rats (SLC, Hamamatsu, Japan) were used (8–24 weeks old) in this study.

### 2.2 Animal surgeries for brain injections

For the injection of neural tracers and AAVs, rats were anesthetized via subcutaneous administration of a combination anesthetic consisting of medetomidine (0.3 mg/kg), midazolam (4.0 mg/kg), and butorphanol (5.0 mg/kg), hereafter referred to as the MMB anesthetic. This protocol reliably induced anesthesia for approximately 1 h. For procedures exceeding 1 h, a half-dose of the MMB anesthetic was administered every 50 min. Anesthetic depth was continuously monitored by assessing body temperature and respiration rate. Body temperature was maintained at 38°C using a heating pad and rectal temperature sensor (BWT 100A animal warmer; Bio Research Center, Nagoya, Japan). The fur on the scalp was trimmed, and local anesthesia (Xylocaine Gelee; Sandoz K.K., Tokyo, Japan) was applied to the skin. The skin was incised with scissors to expose the skull. The nose bar was adjusted to align the bregma and lambda horizontally. Small craniotomies were made using a dental drill to expose the dura mater. Injection coordinates were determined based on the rat brain atlas (Paxinos and Watson, 2013) as follows: GPe [−1.3 mm caudal to the bregma (A −1.3), 2.9 mm lateral from the midline (L 2.9), 5.4 mm deep from the cortical surface (D 5.4)]; lateral striatum [A 1.5, L 3.8, D 3.6]; subthalamic nucleus [A −3.5, L 2.6, D 7.6]; ventral thalamus including VA, VL, and VM [A −2.1 to −3.1, L 2.0, D 5.8–6.8]; primary motor cortex [A 2.0, L 2.6, D 1.0]; secondary motor cortex [A 4.0, L 1.7, D 1.0]. Rats were used for morphological and/or electrophysiological experiments more than 2 weeks after the final AAV injection. In cases involving dual AAV injections, the second AAV was administered 1 week after the first. AAVs used in this study were purchased from Addgene (Watertown, MA, United States), as listed in Table 1. For retrograde tracer experiments, a 5% solution of FluoroGold (FG; Fluorochrome, Denver, CO, United States) or a 0.2% solution of cholera toxin subunit B conjugated to Alexa Fluor 555 (CTB555) or 488 (CTB488) (C22843 or C-22841; Thermo Fisher Scientific, Waltham, MA, United States) dissolved in 0.1 M phosphate-buffered saline (PBS, pH 7.4) was injected at least 4 days prior to perfusion. Each tracer was loaded into a glass micropipette (tip diameter: 20 μm) and inserted into the brain. After a 5-min stabilization period at the targeted site, tracers were delivered by repetitive air pulses (15 psi, 5 ms per pulse) using a picopump (PV820; World Precision Instruments, Sarasota, FL, United States). Following injection, the micropipette was left in place for 10 min before withdrawal. The craniotomy was sealed with bone wax (Ethicon Inc., Raritan, NJ, United States), and the skull was rinsed with sterile saline. The incision was sutured and disinfected with povidone-iodine solution. After recovery from anesthesia, the rats were returned to their home cages.

**TABLE 1:** AAVs used in this study.

Name	Company/ID	References	Titer (vg/mL)	RRID
AAVdj-ReaChR-citrine	Addgene/50954	(Lin et al., 2013)	7 × 10^12^	Addgene_50954
AAVdj-flex-ReaChR-citrine	Addgene/50955	(Lin et al., 2013)	1 × 10^12^	Addgene_50955
AAVrg-pmSyn1-EBFP-Cre (AAV Retro)	Addgene/51507	(Madisen et al., 2015)	7 × 10^12^	Addgene_51507
pENN-AAV1-hSyn-Cre-WPRE-hGH	Addgene/105553	–	7 × 10^12^	Addgene_105553
AAV1-EF1a-Flpo	Addgene/55637	(Fenno et al., 2014)	7 × 10^12^	Addgene_55637
AAV1-Ef1a-fDIO-tdTomato	Addgene/128434	(Sciolino et al., 2022)	1 × 10^13^	Addgene_128434
AAV1-Ef1a-fDIO EYFP	Addgene/55641	(Fenno et al., 2014)	7 × 10^12^	Addgene_55641

### 2.3 *Ex vivo* electrophysiological recording and data analysis

#### 2.3.1 Recording

Basal ganglia neurons were recorded using *ex vivo* whole-cell patch-clamp techniques as previously described (Karube et al., 2019). Male Wistar rats (*N* = 24 rats; postnatal day 30–65) were deeply anesthetized with isoflurane and transcardially perfused with 25 mL of ice-cold modified artificial cerebrospinal fluid (ACSF) containing (in mM): N-methyl-D-glucamine, 93; KCl, 2.5; NaH_2_PO_4_, 1.2; NaHCO_3_, 30; HEPES, 20; glucose, 25; sodium ascorbate, 5; thiourea, 2; sodium pyruvate, 3; MgCl_2_, 10; and CaCl_2_, 0.5. The pH was adjusted to 7.3 with HCl. All ACSF solutions were continuously bubbled with 95 O_2_ and 5% CO_2_. Brains were quickly removed and immersed in ice-cold modified ACSF for 2 min. Coronal slices (300 μm thick) were cut using a vibratome (7,000 smz-2; Campden Instruments, Leicestershire, United Kingdom) and incubated in modified ACSF at 32°C for 15 min. Slices were then transferred to normal ACSF containing (in mM): NaCl, 125; KCl, 2.5; CaCl_2_, 2.4; MgCl_2_, 1.2; NaHCO_3_, 25; glucose, 15; NaH_2_PO_4_, 1.25; pyruvic acid, 2; lactic acid, 4, and maintained at room temperature. After 1 h of recovery, slices were placed in a recording chamber maintained at 30°C. Whole-cell recordings were performed using borosilicate glass pipettes (4–6 MΩ) filled with intracellular solution based on either KCl, CsCl, or K-gluconate. The compositions are as follows: KCl-based solution (in mM): KCl, 135; NaCl, 3.6; Na_2_ATP, 2; NaGTP, 0.4; MgCl_2_, 1; Na_4_EGTA, 0.5; HEPES, 10; biocytin, 20.1; CsCl-based solution: CsCl, 120; tetraethylammonium-Cl, 5; Na_2_ATP, 2; NaGTP, 0.5; Na_4_EGTA, 0.25; HEPES, 5; QX-314-Cl, 0.5; biocytin, 20.1; K-gluconate-based solution: K-gluconate, 130; KCl, 2; Na_2_ATP, 3; NaGTP, 0.3; MgCl_2_, 2; Na_4_EGTA, 0.6; HEPES, 10; biocytin, 20.1. The pH was adjusted to 7.3 with KOH or CsOH, and the osmolality was adjusted to ∼290 mOsm. Target brain regions were identified using a fluorescence microscope (BX-51WI; Olympus, Tokyo, Japan) equipped with a 40 × water-immersion objective. Voltage- and current-clamp recordings were low-pass filtered at 10 kHz and digitized at 20 kHz using an EPC10 amplifier (HEKA Elektronik Dr. Schulze GmbH, Lambrecht/Pfalz, Germany). Series resistance was monitored by applying a −10 mV voltage pulse for 10 ms and confirmed to be < 25 MΩ throughout the recording. Within 1 min of achieving whole-cell configuration, firing responses to 1-s depolarizing current pulses (maximum 1,000 pA, incremented in 50 pA steps) were recorded in current-clamp mode when using K-gluconate or KCl-pipette solution. Passive membrane properties were assessed using 1-s hyperpolarizing current pulses. Putative MSNs and cholinergic interneurons (CIN) were distinguishable by their characteristic morphology and firing patterns. For photoactivation of channelrhodopsin-2 (ChR2), a 470 nm LED (BLS-LCS-0470-50-22, Mightex Systems, Pleasanton, CA, United States) was used to deliver full-field illumination through a 40 × water-immersion objective. Blue light pulses (5 ms duration, ∼4 mW total power) were delivered at 10 Hz for 1 s (10 pulses) to optically stimulate ChR2-expressing axon terminals. This stimulation protocol was repeated 10–15 times at 1-s intervals. In some experiments, low concentrations of tetrodotoxin (TTX, 1 μM) and 4-amino pyridine (100 μM) were added to the ACSF to isolate monosynaptic currents (Petreanu et al., 2009; Shu et al., 2007). DNQX (10 μM) and AP5 (50 μM) were applied to block glutamatergic transmission, and SR95531 (Gabazine; 20 μM) was applied to inhibit GABA_*A*_ receptor-mediated synaptic currents. All pharmacological reagents were purchased from Tocris Bioscience (Bristol, United Kingdom).

#### 2.3.2 Electrophysiological data analysis

The analysis method has been previously described (Karube et al., 2019). Briefly, recordings were analyzed using Igor Pro 9 (WaveMetrics Inc., Portland, OR) and the Neuromatic plugin^1^ (Rothman and Silver, 2018) and custom-built procedures. Input resistance was determined via the linear fitting of voltage responses to hyperpolarized current pulses (from −20 to −100 pA, in 20 pA increments). The membrane time constant was calculated from the voltage response to a −50 pA current pulse. To identify optically evoked inhibitory postsynaptic currents (oIPSCs), each trace was smoothed using a 0.2-ms moving average (4 consecutive data points), and all traces were aligned to the onset of photo stimulation to calculate the average response. Baseline was defined as the mean current over the 50 ms preceding photo stimulation. Amplitude was measured from baseline to peak of the current with a stable delay after stimulation onset. Inward currents were classified as oIPSCs if their peak amplitude exceeded three times the standard deviation of the baseline. The rising phase of the oIPSC was linearly fitted and extrapolated to determine the intersection with the baseline, which was defined as the onset of the oIPSC. Latency was defined as the interval between photo stimulation onset and oIPSC onset.

#### 2.3.3 Post-recording tissue processing

Following electrophysiological recordings, slices were fixed overnight at 4°C in a solution containing 4% paraformaldehyde, 0.05% glutaraldehyde, and 0.2% picric acid in 0.1 M phosphate buffer (PB). Fixed slices were rinsed three times with PB (10 min each). In some cases, slices were re-sectioned into 50 μm-thick sections using a vibratome. To quench endogenous peroxidase activity, sections were incubated in 1% H_2_O_2_ in PB for 30 min at room temperature, followed by three rinses in PB. For fluorescent visualization of biocytin-filled neurons, sections were incubated with CF350-conjugated streptavidin (1:3,000; Biotium, Inc, Fremont, CA) for 2 h at room temperature. For immunohistochemical detection of choline acetyl transferase (ChAT) or choline transporter I (CHT1), slices were processed using the CUBIC tissue-clearing protocol (Susaki et al., 2015, 2020). Briefly, slices were cryoprotected by sequential incubation in 15 and 30% sucrose in PB (3 h each). Followed by two freeze-thaw cycles using dry ice. Slices were then incubated overnight in CUBIC-1 solution, composed of 25% urea, 25% N,N,N’,N’-tetrakis-(2-hydroxypropyl)-ethylenediamine, and 15% polyethylene glycol mono-p-isooctylphenyl ether in distilled water. After washing with PBS containing 0.3% Triton X (PBS-X), slices were incubated overnight (1–2 nights) with primary antibody solution against ChAT or CHT1 diluted in incubation buffer containing 10% normal donkey serum, 2% bovine serum albumin, 0.02% sodium azide, and 0.5% Triton X in 0.05 M tris-buffered saline (TBS). After three rinses with PBS-X (30 min each), slices were incubated in a secondary antibody mixture containing CF350-conjugated streptavidin (1:2,000 diluted) and additional secondary antibodies solved in incubation buffer. Following additional washing, slices were incubated overnight in CUBIC-2 solution (10% 2,2′,2″-nitrilotriethanol, 50% sucrose, and 25% urea in distilled water). Cleared sections were imaged using confocal microscopy (FV1200; Olympus) equipped with a long working distance objective (×100 silicon immersion lens) to visualize biocytin-filled neurons. For brightfield microscopy, sections were incubated overnight at 4°C in avidin-biotin complex (VECTASTAIN Elite ABC kit; 1:200; Vector laboratories, Newark, CA, United States). Biocytin-filled neurons were visualized using nickel-enhanced diaminobenzidine in the presence of H_2_O_2_ at a final concentration of 0.01%. Sections were air-dried on glass slides and coverslipped using EcoMount (Biocare Medical, LLC, Concord, CA) or Mount-Quick (Daido Sangyo, Toda, Japan).

### 2.4 Histology, image acquisition and analysis

#### 2.4.1 Histology

Transcardial perfusion and tissue processing were performed as previously described (Karube et al., 2019). Briefly, rats were deeply anesthetized and perfused with a pre-fixative solution. Following deep anesthetization with an overdose of isoflurane or intraperitoneal administration of pentobarbital and perfused transcardially with a pre-fixative solution containing 50 mM MgCl_2_ and 7.5% sucrose in 0.02 M PB. This was followed by fixation with 4% paraformaldehyde and 0.2% picric acid (both from Nacalai Tesque, Tokyo, Japan) in 0.1 M PB (pH 7.4). Rats were postfixed *in situ* for 2–3 h at room temperature. Brains were then removed and rinsed three times with 0.1 M PB (30 min each) and subsequently soaked in 30% sucrose in 0.1 M PB until they sank. Brains were sectioned at 30–40 μm thickness using a freezing microtome (SM2000R; Leica Microsystems, Wetzlar, Germany). Sections were stored in 0.1 M PB containing 0.02% sodium azide at 4°C until use.

For immunostaining, sections were incubated with the primary antibody solution diluted in incubation buffer for 1–2 overnights at room temperature. After three rinses with TBS, sections were incubated with the secondary antibodies (Jackson ImmunoResearch, West Grove, PA, United States; Thermo Fisher Scientific, Waltham, MA, United States) diluted in the same incubation buffer for 3 h at room temperature. Sections were rinsed three times with TBS (10 min each), mounted on glass slides, air-dried, and covered using Prolong Gold antifade reagents (Invitrogen, Thermo Fisher Scientific). Antibodies used in this study are listed in Table 2.

**TABLE 2:** Antibodies and reagents list.

Antigen	Species	Company/Catalog #	Dilution	RRID
Calbindin	Mouse	Swant/CB-38a	1:4,000	AB_3107026
Choline acetyltransferase	Mouse	Millipore/MAB305	1:500	AB_11212978
Choline acetyltransferase	Goat	Millipore/AB144P	1:500	AB_90650
CHT1	Goat	Nittobo Medical/CHT-Go-Af890	1:4,000	AB_2721226
Cre	Guinea pig	Synaptic Systems/257005	1:1,000	AB_2943537
Cre	Mouse	Millipor/MAB3120	1:500	AB_94033
FoxP2	Rabbit	Abcam/ab16046	1:2,000	AB_443473
FoxP2	Mouse	Millipore/MABE4	1:2,000	AB_10805892
Gephyrin	Mouse	Synaptic systems/147008	1:1,000	AB_887722
GFP	Chicken	GeneTex/GTX13970	1:1,000	AB_371416
Mu-opioid receptor	Rabbit	Neuromics/RA10104	1:1,000	AB_2230500
Nkx2.1	Mouse	Novocastra/NCL-L-TTF1	1:1,000	AB_442193
Nkx2.1	Rabbit	Millipore/07-601	1:5,000	AB_310305
Parvalbumin	Rabbit	Swant/PV25	1:5,000	AB_10000344
Parvalbumin	Guinea pig	Synaptic systems/195308	1:4,000	AB_2571613
TdTomato	Goat	Lifespan Biosciences/LS-C340696	1:1,000	AB_2819022
TdTomato	Rat	Kerafast/16D7	1:10,000	AB_2754715
Vesicular glutamate transporter 1	Goat	Nittobo Medical/VGluT1-Go-Af310	1:500	AB_2571617
Vesicular glutamate transporter 2	Guinea pig	Nittobo Medical/VGluT2-GP-Af810	1:100	AB_2571621
Vesicular GABA transporter	Rabbit	Nittobo/VGAT-Rb-Af500	1:1,000	AB_2571646

#### 2.4.2 Image acquisition and analysis

Fluorescent images were acquired using epifluorescent microscopes (BX53; Olympus) equipped with an Orca Spark CMOS camera (Hamamatsu photonics, Hamamatsu, Japan) or a BZ-700 microscope system (Keyence, Tokyo, JAPAN). For high-resolution images, a confocal microscope (FV1200) was used with 40 × [numerical aperture (N.A.) 0.95), 60 × (N.A. 1.40) or 100 × (N.A. 1.35; silicon oil immersion] objectives. Brightfield photomicrographs were captured using a CCD camera (DP-73, Olympus) mounted on a BX-53 microscope with 4 × (N.A. 0.13), 10 × (N.A. 0.3), and 40 × (N.A. 0.75) objectives. Digitized images were analyzed using Fiji (a distribution of ImageJ) (Schindelin et al., 2012). Image brightness was adjusted using the “adjust levels” function. To generate multi-focus composite images, z-stack images were captured at 0.2 μm (for axonal varicosities) or 2 μm (for cell bodies and dendrites) intervals and processed using the “extended depth of focus” plugin in Fiji. Neurons and axonal varicosities were manually counted. For comparison of fluorescent intensity between striatal striosome and matrix compartments, discrete MOR-positive striosome islands were delineated using binarized images and thresholding functions, and their areas were measured. A region of interest (ROI) of equal area was placed in the adjacent MOR-negative matrix compartment. These striosome-matrix ROI pairs were used to calculate relative pixel intensity. To compare distribution of labeled structures (varicosities or neurons) between the matrix and striosome compartments, striosome-matrix ROI pairs were assigned as described above, and labeled structures within each ROI were manually counted.

### 2.5 Statistical comparisons

Statistical analyses were performed using R software^2^ (R Project for Statistical Computing, Vienna, Austria) and Microsoft Excel. Averaged data are presented as mean ± standard deviation unless otherwise noted. Comparisons among more than two groups were conducted using one-way ANOVA followed by *post hoc* Tukey tests. Comparisons between two groups were performed using the Wilcoxon rank-sum test. To test whether the mean relative value significantly differed from 1 (null hypothesis), the Wilcoxon signed-rank test was applied. Statistical significance was defined as *p* < 0.05. Significant differences are indicated by asterisks (*p < 0.05; **p < 0.01; ***p < 0.001). All *p*-values are reported.

## 3 Results

### 3.1 GPe projections target the matrix compartment of the striatum

**FIGURE 1:**
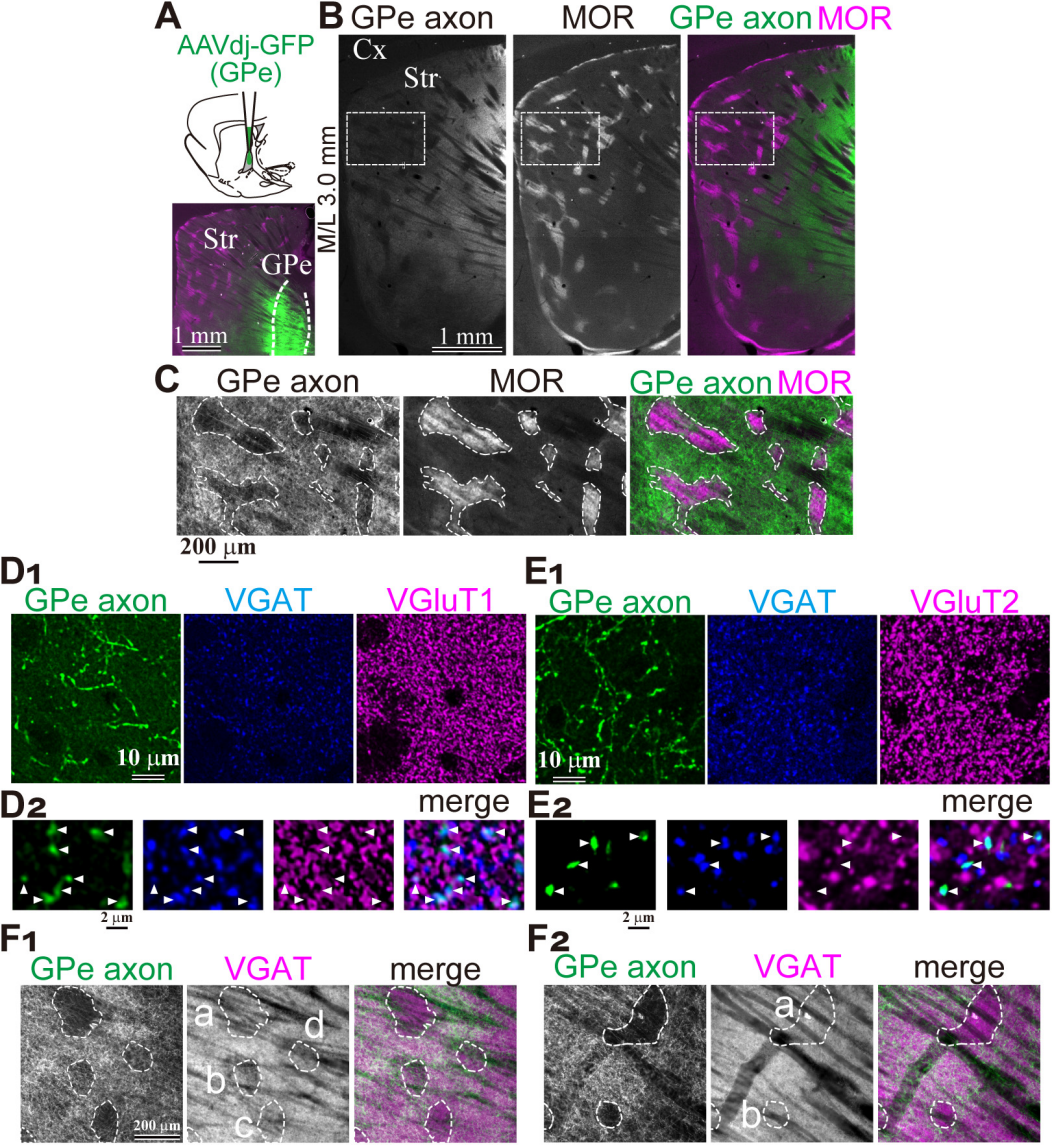
Preferential projection of GPe axons to the striatal matrix compartment. **(A)** Experimental overview. Top, Schematic illustration of AAV injection into the GPe. Bottom, Representative injection site in the GPe. **(B)** Distribution of GPe axons in the striatum. Left, AAV-labeled axons. Middle, Immunostaining for mu-opioid receptor (MOR). Right, Merged image showing AAV-labeled axons (green) and MOR expression (magenta). **(C)** Higher magnification images of AAV-labeled axons (green) and MOR labeling (magenta) in the boxed area shown in B. MOR-positive striosome compartment is delineated (dotted line). Note the highly preferential axonal projection to the MOR-negative matrix compartment. **(D,E)** Confirmation of the GABAergic nature of AAV-labeled axons using immunostaining for VGAT (blue) and either VGluT1 (magenta in **D**) or VGluT2 (magenta; **E**). **(D1,E1)** Low magnification images showing overall distribution. **(D2,E2)** High magnification confocal images. Note axonal varicosities (arrowheads) colocalized with VGAT, but not with VGluT1 **(D2)** or VGluT2 **(E2)**. **(F)** Heterogeneous spatial relationship between GPe axonal territories and VGAT expression. **(F1)** Left: Regions (a-d) lacking GPe axonal projections are delineated by dotted lines. Middle: These regions (a-d) exhibited relatively weak VGAT expression. Right: Merged view of GPe axons (green) and VGAT signals (magenta). **(F2)** Similar images from a different striatal area in the same animal shown in F1. VGAT expression levels in the regions with less GPe axons (a, b) remain comparable to neighboring regions with dense GPe projections in this area. Cx, cerebral cortex; GPe, globus pallidus external segment; Str, striatum.

To analyze GPe axons in the striatum, AAV-ReaCh-Citrine was injected into the rat GPe (*N* = 3 rats; Figure 1A). Fluorescently labeled axons were clearly detected across broad areas of the striatum, interspersed with small islands of weak fluorescence that formed patch-like regions. Immunostaining for mu-opioid receptors (MOR) revealed that these weakly fluorescent islands corresponded to regions of high MOR expression, identifying them as the striosome compartment (Figure 1B). Magnified views demonstrated a complementary relationship between MOR expression and the distribution density of GPe axons (Figure 1C). However, it should be noted that axons originating from brain regions other than the GPe may pass through the GPe and be inadvertently labeled by the AAV, potentially confounding the results. In particular, contamination by thalamic axons expressing VGluT2 is a concern, as VGluT2 expression has been reported to be significantly higher within the matrix compartment than in the striosome.

To assess potential contamination, we performed double immunostaining for vesicular GABA transporter (VGAT) and either vesicular glutamate transporter 1 (VGluT1) or VGluT2 in striatal sections (Figure 1D1). Manual counting of GFP-labeled axonal varicosities revealed that 210 of 217 were VGAT-positive, with no detectable VGluT1 expression (0/217) (Figure 1D2). Similarly, VGAT was expressed in 195 of 209 varicosities, while VGluT2 was detected in only 1 of 209 (Figures 1E1,E2). These results indicate that the AAV-labeled axons were GABAergic, with minimal contamination from glutamatergic axons, and were most likely to originate from the GPe. However, it should be noted that VGAT expression in the striosome was not uniform throughout the striatum (Figure 1F). VGAT expression was indeed weak in some striosomes, although comparable to that in the matrix in others. This suggests that distinct GABAergic innervation may govern the matrix and striosome compartments, potentially reflecting heterogeneous origins.

**FIGURE 2:**
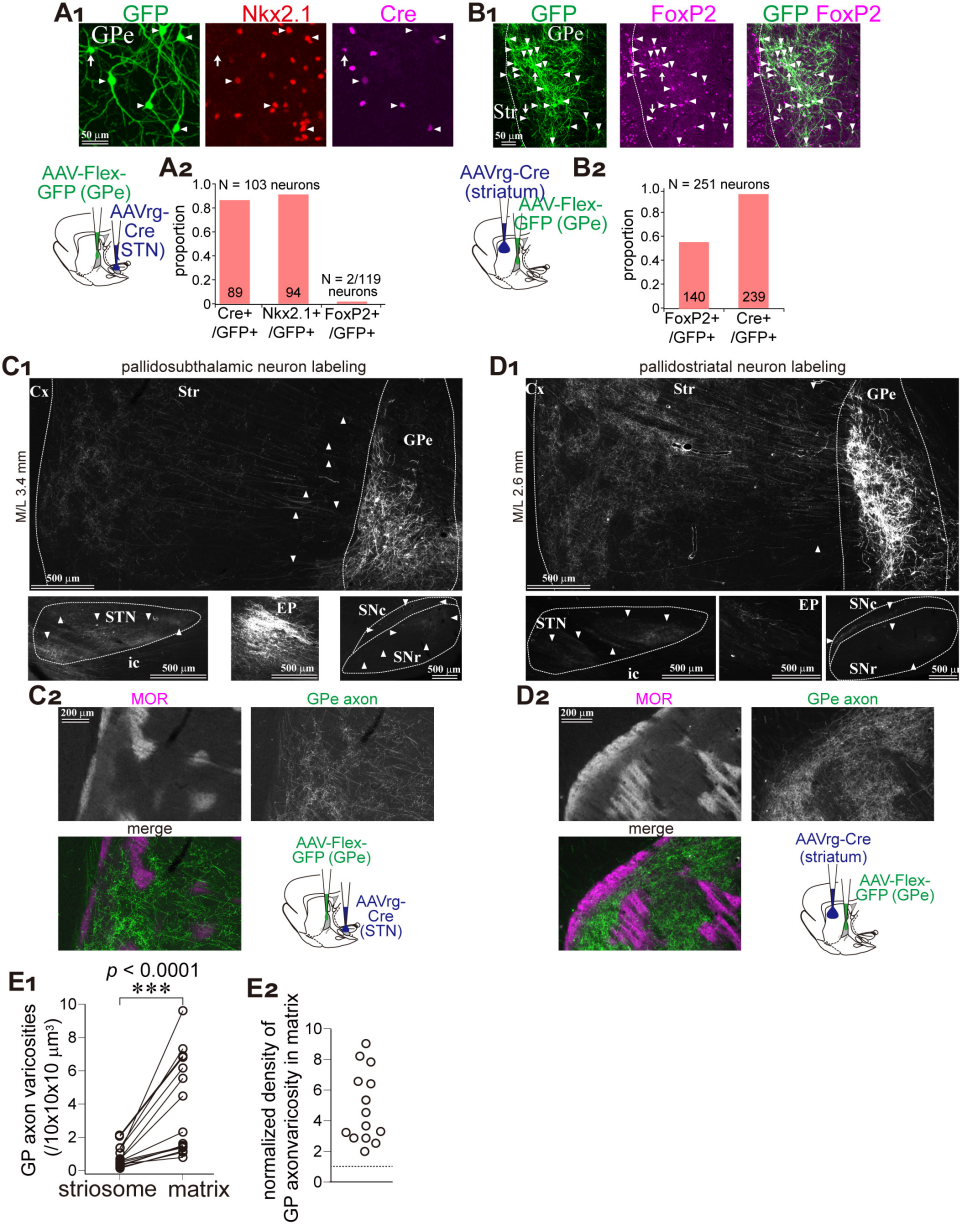
Matrix-preferential GPe axonal projections are independent of neuron subtypes. **(A)** Prototypic GPe neurons were selectively labeled using a combination of retrograde AAV (AAVrg-Cre) and Cre-dependent AAV-Flex-GFP (schematic shown in lower left). GFP-labeled neurons (green; left, arrowheads) coexpressed Nkx2.1 (red; middle), a marker of prototypic neurons, and Cre (magenta; right). **(A2)** Quantification of coexpression between GFP and either Cre, Nkx2.1, or FoxP2 (a marker of arkypallidal neurons). **(B)** AAV-mediated labeling of striatum-projecting GPe neurons (illustrated in a lower left panel). **(B1)** GFP-labeled neurons (green; left, arrowheads) frequently coexpressed FoxP2 (magenta; middle). Merged image is shown on the right. **(B2)** More than half of GFP-labeled neurons were FoxP2-positive. **(C,D)** Axonal distribution of prototypic **(C)** and striatum-projecting **(D)** GPe neurons. **(C1,D1)** GFP expression in GPe (top), STN (lower left), EP (lower middle), and SN (lower right). Axons to the striatum can be followed (arrowheads in top). Striatum-projecting GPe neurons exhibited denser projections to the striatum, whereas prototypic neurons projected densely to STN, EP, and SN. **(C2,D2)** High magnification images of GFP-labeled axons in the striatum. MOR immunostaining revealed that both types of labeling resulted in predominant projection to the matrix. **(E)** Quantitative comparison of GFP-labeled viscosity density between matrix and striosome compartments. **(E1)** Density measurements from paired striosome and matrix regions of equal area. **(E2)** Ratio of the density in matrix relative to striosome, indicating high preference to matrix.

The GPe contains two types of projection neurons: prototypic neurons, which primarily project to the STN with minor collateral branches to the striatum, and arkypallidal neurons, which project exclusively to the striatum without downstream collaterals. To label GPe neurons in a cell-type-selective manner, we employed retrograde AAV injections into either the striatum or the STN, followed by Cre-dependent GFP expression via a second AAV injection into the GPe (Figures 2A,B; *N* = 3 rats each). Following AAVrg injection into the STN, 91.3% of GFP-labeled GPe neurons expressed NKx2.1 (N = 94 of 103), a marker of prototypic neurons, whereas only 1.7% expressed FoxP2, which marks arkypallidal neurons (Figure 2A). This minor FoxP2 expression may be attributable to rare coexpression between PV and FoxP2 in rats (Abdi et al., 2015). In contrast, AAVrg injection into the striatum preferentially labeled FoxP2-positive GPe neurons (55.8%; *N* = 140 of 251; Figure 2B). Thus, prototypic neurons were selectively labeled via STN injection, while arkypallidal neurons were labeled specifically via striatal injection.

We analyzed the distribution of GFP-labeled GPe axons in the striatum (Figures 2C,D). Although axonal density in the striatum was higher in samples labeled via striatal injection, a clear matrix preference was observed in both labeling conditions (Figures 2C2,D2). In addition, labeled axons in downstream basal ganglia structures—STN, EP, and SN—were markedly reduced in the striatum-injected samples, reflecting a reduced contribution from prototypic neurons (Figures 2C1,D1). We quantified the number of GPe axonal varicosities in striosome islands and adjacent matrix regions of equal area (Figure 2E1; *N* = 3 rats; total of 8037 varicosities across 15 ROIs per compartment). On average, 0.83 ± 0.64 varicosities per 10 × 10 × 10 μm^3^ were found in the striosome, and 3.86 ± 2.94 per 10 × 10 × 10 μm^3^ in the matrix, indicating a significant difference (*p* = 6.1 × 10^5^, Wilcoxon signed-rank exact test). Since varicosity counts varied widely among ROIs, we also calculated the ratio of varicosity density in the matrix relative to the paired striosome (Figure 2E2). The average ratio was 4.92 ± 2.26 (range: 1.99–9.04). These data suggest that matrix-selective innervation by GPe neurons is a common property, irrespective of neuronal subtype.

### 3.2 GPe inputs to striatal neurons

**FIGURE 3:**
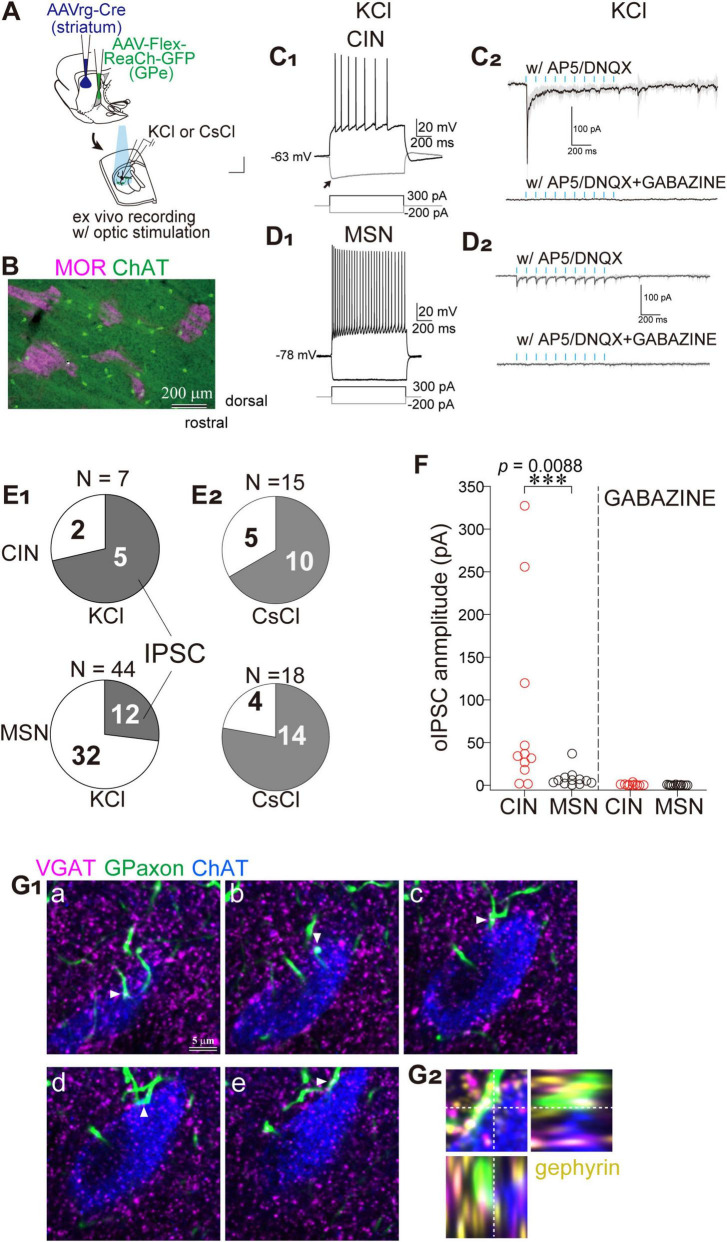
Preferential innervation of striatal cholinergic neurons (CINs) by GPe axons within matrix compartments. **(A)** Schematic illustration of AAV injections and whole cell recordings. **(B)** Double immunostaining for ChAT (green) and MOR (magenta) reveals that CINs are predominantly located within matrix compartments. **(C,D)** Representative whole-cell recordings from CINs **(C)** and MSNs **(D)** using KCl-based pipette solution. **(C1,D1)** Current-clamp recordings show a relatively depolarized resting membrane potential in CINs (- 63 mV; **C1**), compared to a more hyperpolarized potential in MSNs (- 78 mV in **D1**). Voltage responses to hyperpolarizing and depolarizing current injections demonstrate cell-type-specific electrophysiological properties. **(C2,D2)** Voltage-clamp recordings at - 60 mV from CINs **(C2)** and MSNs **(D2)** during optical stimulation of GPe axon terminals (cyan ticks), in the presence of glutamate receptor antagonists (AP5 and DNQX; top), and with additional GABA_*A*_ receptor blockade by Gabazine (bottom). Averaged traces (solid lines) and standard deviation (gray shading) from 10 consecutive sweeps are shown. Note the prominent initial inward current in CINs. **(E)** Connection probability of GPe axons to CINs (top) and MSNs (bottom). **(E1)** Recordings with KCl-based pipette solution. **(E2)** Recordings with CsCl-based pipette solution. Shaded area indicates a fraction of neurons exhibiting IPSCs. **(F)** Summary of IPSC amplitude recorded in CINs (red circles) and MSNs (black circles) using CsCl-based pipette solution. The IPSCs were almost abolished by the application of Gabazine. **(G)** Subcellular localization of putative synaptic contacts on a CIN. **(G1)** Confocal images of five optical sections (a-e) from a single CIN stained for ChAT (blue), GPe axons (green), VGAT (magenta), and gephyrin (yellow; omitted in **G1** to maintain visibility; shown in **G2**). VGAT-expressing GPe axonal varicosities are apposed to the soma and proximal dendrites of the CIN (arrowheads). **(G2)** Orthogonal views of the apposition shown in G1d.

Using whole-cell patch-clamp recordings combined with optogenetics, we analyzed synaptic connections between GPe axons and identified striatal neurons. To record GABAergic synaptic currents as inward currents, a high-concentration KCl or CsCl pipette solution was used (Figure 3A; see Materials and methods). Recordings were performed in GPe axon-rich regions, specifically within the matrix compartment of the dorsolateral striatum.

Since cholinergic interneurons (CINs) are preferentially distributed in the matrix compartment compared to the striosome (Figure 3B; Kubota and Kawaguchi, 1993), we hypothesized that they may serve as preferential targets of GPe axons. To identify putative CINs, cells with large somata were selected and subsequently confirmed via *post hoc* visualization of the recorded neurons, combined with immunofluorescent detection of choline acetyltransferase (ChAT) or choline transporter 1 (CHT1). Voltage responses to current injections were recorded in current-clamp mode, which enabled reliable cell-type classification. As previously reported (Kawaguchi, 1992), CINs exhibited accommodating and slow action potentials in response to depolarizing current pulses (Figure 3C1). Additionally, a hyperpolarizing sag potential was elicited in response to negative current injection (Figure 3C1). Optically evoked synaptic currents were recorded in voltage-clamp mode using a high-concentration KCl-based pipette solution. Blue light stimulation (5-ms pulses at 10 Hz) induced large inward currents in CINs in the presence of glutamate receptor antagonists AP5 and DNQX (Figure 3C2). The first pulse evoked the largest response, with subsequent pulses producing incrementally smaller currents. The currents were confirmed as optically evoked inhibitory postsynaptic currents (oIPSCs) mediated by GABA_*A*_ receptors, as they were completely abolished by application of Gabazine (Figure 3C2). In contrast, MSNs exhibited more hyperpolarized resting membrane potentials (Figure 3D1). Depolarizing current pulses induced non-accommodating high-frequency firing in MSNs (Figure 3D1). The oIPSCs recorded in MSNs were relatively small, although repetitive light stimulation consistently elicited responses (Figure 3D2). CINs were frequently innervated by GPe axons (5 of 7 neurons), whereas the connection probability between GPe axons and MSNs was low (12 of 44 neurons) (Figure 3E1). These differences may be attributed to cell type-dependent connectivity or, alternatively, to cell type-specific subcellular synapse localization. To investigate these possibilities, a CsCl-based pipette solution was used, which enables recording of oIPSCs arising from distal dendritic compartments. The connection probability in CINs was comparable between KCl and CsCl conditions (5 of 7 with KCl vs. 10 of15 with CsCl). However, in MSNs, the connection probability markedly increased with CsCl (14 of 18 vs. 12 of 44 with KCl; Figure 3E2). Despite this increase, the amplitude of oIPSCs remained substantially smaller in MSNs than in CINs (Figure 3F). Under CsCl conditions, the amplitude of oIPSC was 7.54 ± 9.41 pA in MSNs (*N* = 6 mice, 13 neurons) and 81.85 ± 109.50 pA in CINs (*N* = 8 mice, 11 neurons) in the presence of AP5 and DNQX, indicating a significant difference (*p* = 0.008, Wilcoxon rank sum test). Following Gabazine application, oIPSC amplitude was reduced to 0.20 ± 0.33 pA in MSNs and 0.67 ± 1.09 pA in CINs, with no statistically significant difference (*p* = 0.26).

To confirm the subcellular localization of putative synapses, appositions of GPe axons onto CINs were examined using immunohistochemical labeling for choline acetyltransferase (ChAT), vesicular GABA transporter (VGAT), and gephyrin, a marker of postsynaptic GABAergic structures (Figure 3G). Confocal imaging revealed frequent appositions of GPe axons in close proximity to cell bodies and proximal dendrites of CINs. Moreover, VGAT and gephyrin were co-localized at GPe terminals and on CINs, respectively, supporting the presence of functional GABAergic synapses (Figure 3G). These results suggest that the biased innervation of GPe axons to the striatal matrix compartment may elicit robust inhibitory control over CINs.

### 3.3 Reciprocal relationship between striatal compartments and GPe

**FIGURE 4:**
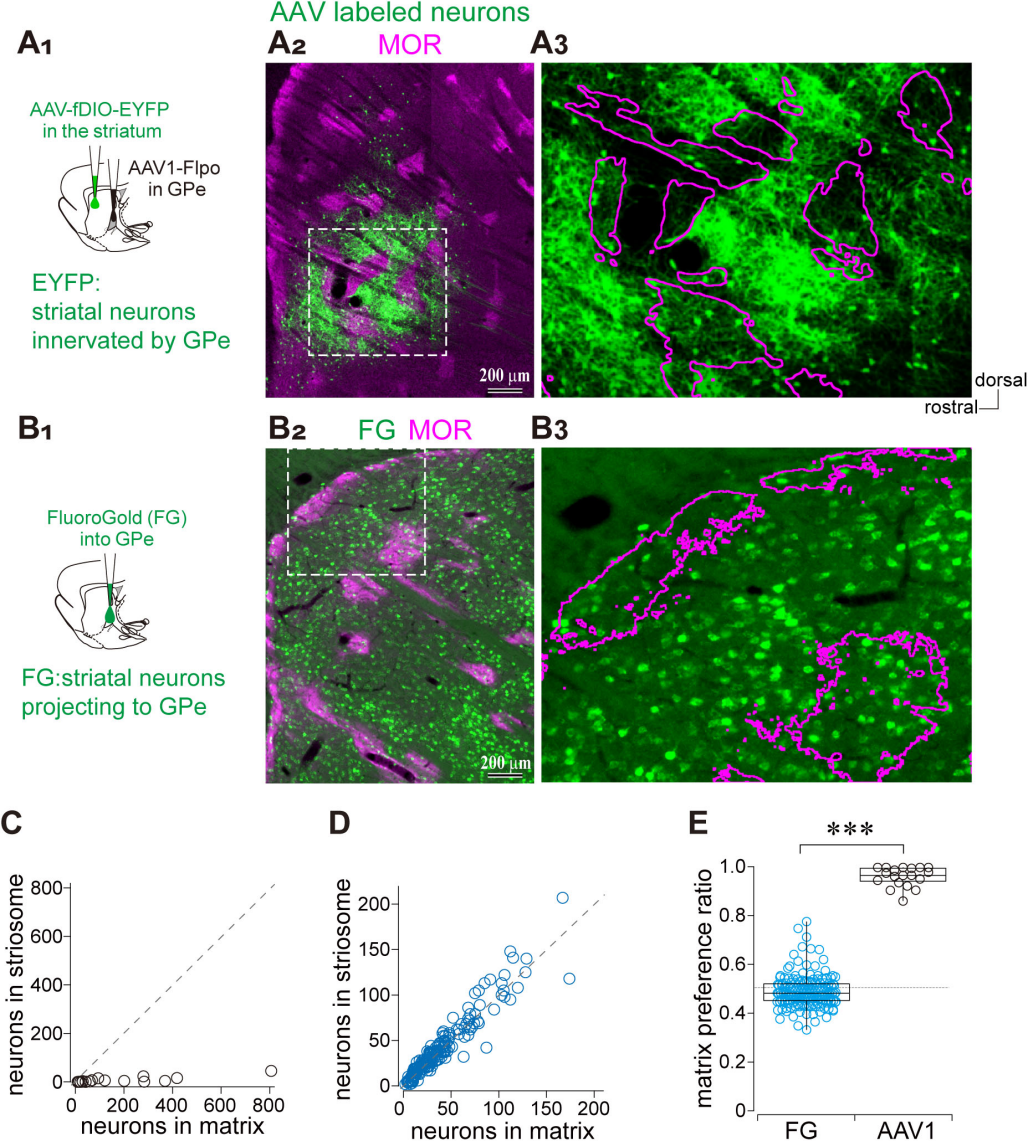
Asymmetric parallel projections between the GPe and striatal compartments. **(A)** Visualization of striatal neurons receiving GPe inputs using AAV1. **(A1)** Schematic illustration of AAV injections. **(A2)** Low-magnification image showing the distribution of AAV-labeled striatal neurons (green) and MOR immunostaining (magenta). **(A3)** Higher magnification of the boxed region in **(A2)**. Striosome boundaries are indicated by magenta contours. **(B)** Visualization of striatal neurons projecting to the GPe using FluoroGold. **(B1)** Schematic illustration of retrograde tracer (FluoroGold) injection into the GPe. **(B2)** Low-magnification image showing the distribution of FluoroGold-labeled striatal neurons (green) and MOR immunostaining (magenta). **(B3)** Higher magnification of the boxed region in **(B2)**. **(C)** Quantification of AAV-labeled striatal neurons in paired regions of matrix and adjacent striosome of equal area. A dotted line indicates the unity line (equal neuron counts in both compartments). **(D)** Quantification of the FluoroGold labeled striatal neurons in matrix and striosome compartments. **(E)** Box plots showing the matrix preference ratio, which indicates the fraction of labeled neurons located in the matrix compartment relative to the total in both matrix and striosome [i.e., matrix/(matrix + striosome)]. A statistically significant difference was observed (*p* = 0.00006).***p < 0.001.

Given the biased projection from the GPe to the matrix compartment of the striatum, we asked whether the reciprocal projection from the striatum to the GPe also differentiates between matrix and striosome compartments. To address this question, we employed anterograde trans-synaptic labeling by injecting AAV1-hSyn-Flpo into the GPe in combination with AAV-fDIO-GFP into the striatum, enabling visualization of striatal neurons innervated by the GPe (Figure 4A). In parallel, striatal neurons projecting to the GPe were labeled via FluoroGold retrograde tracing from the GPe (Figure 4B). The AAV-labeled striatal neurons were predominantly localized within the matrix compartment, with only a minor fraction found in the striosome (Figures 4A2,A3). We counted the number of GFP-expressing neurons within striosome islands and adjacent matrix regions of equal area (*N* = 3 rats; 19 ROIs per compartment; Figure 4C). In contrast, FluoroGold-labeled striatal neurons projecting to the GPe were distributed uniformly across both matrix and striosome compartments (Figures 4B2,B3). Indeed, the plot of FluoroGold labeled neuron counts in the matrix and striosome revealed that individual data points closely aligned with the unity slope line (*N* = 3 rats; 87 ROIs per compartment; Figure 4D). We further quantified the ratio of labeled neurons in the matrix relative to the total number in both the matrix and striosome compartments (Figure 4E). The proportions were 0.962 ± 0.040 for AAV1-labeled neurons and 0.492 ± 0.066 for FluoroGold-labeled neurons, indicating a highly significant difference (*p* = 8.6 × 10^–13^, Wilcoxon rank-sum test). These findings indicate that the compartmental bias in GPe-striatal connectivity is unidirectional: only the projection from the GPe to the striatum exhibits compartmental selectivity toward the matrix, whereas information from both the matrix and striosome compartments converge onto the GPe.

### 3.4 Distinct excitatory innervation to the matrix compartment

Notably, arkypallidal GPe neurons exhibit low spontaneous activity, whereas prototypic GPe neurons are tonically active (Mallet et al., 2012; Abdi et al., 2015). Therefore, excitatory inputs to arkypallidal neurons may be required to recruit them as an inhibitory source in modulation of striatal neuron activity. We hypothesized that the striatum and GPe may share common excitatory afferents, enabling the GPe–striatum inhibitory pathway to function as a feed forward inhibitory mechanism that regulates the precise timing of striatal neuron activity. It is well established that the major excitatory inputs to the striatum originate from the cerebral cortex and thalamus. Thus, we investigated whether cortical and thalamic projections also provide direct innervation to the GPe. Furthermore, given the selective innervation of matrix compartments by GPe axons, we speculated that these excitatory sources might also exhibit preferential targeting of the matrix compartment. Regarding cortical projections to the GPe, previous studies have demonstrated that motor cortical areas provide direct input to GPe neurons (Karube et al., 2019; Abecassis et al., 2020). Accordingly, we examined the distribution of cortical axons in the striatum originating from the primary and secondary motor cortical areas (M1 and M2, respectively) to assess their compartmental targeting.

**FIGURE 5:**
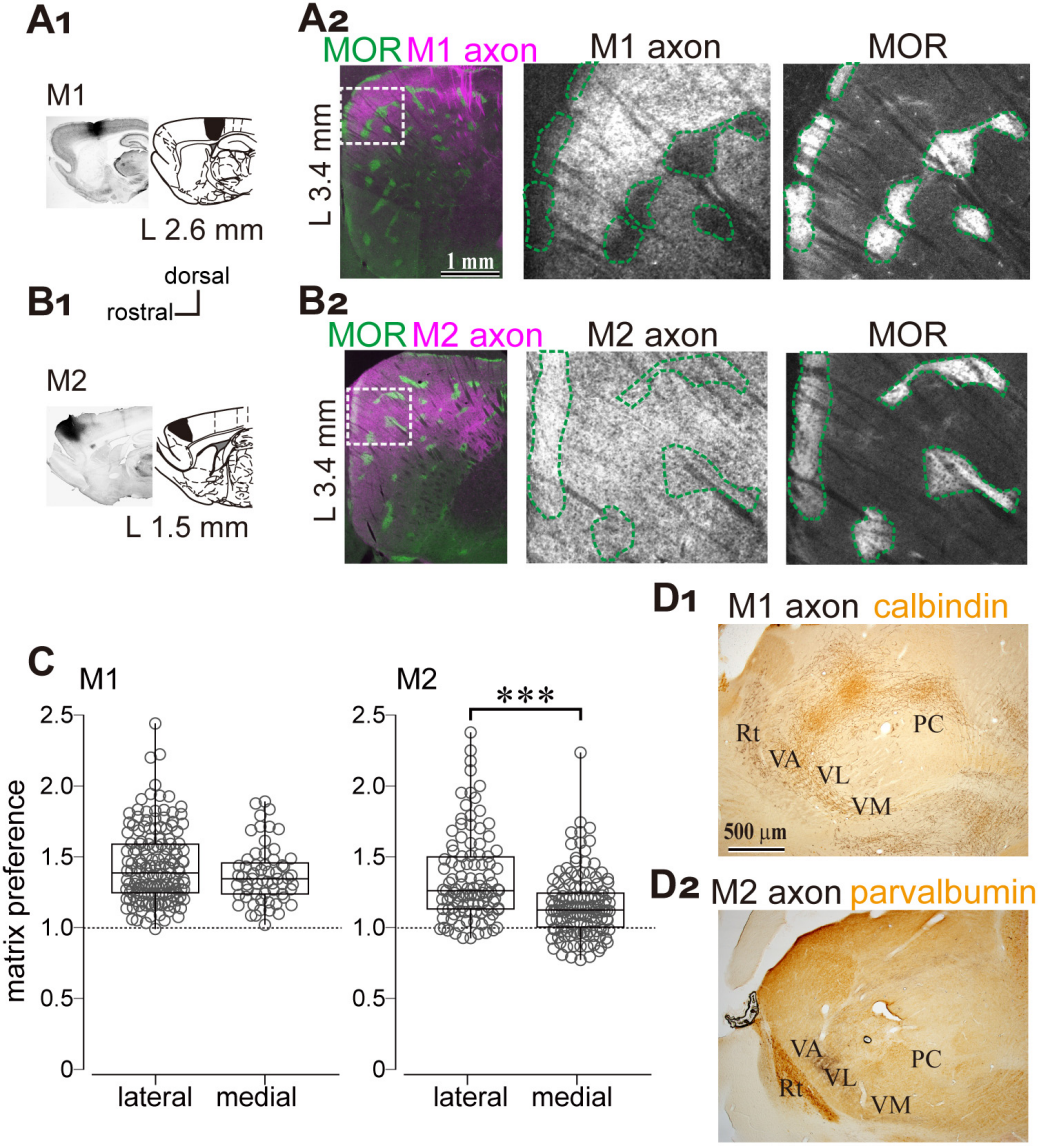
Preferential cortical projections to the striatal matrix compartments from the primary (M1) and secondary (M2) motor cortices. **(A1,B1)** Anterograde tracer injections into M1 **(A1)** and M2 **(B1)**. Left: Representative images of injection sites. Right: Corresponding brain atlas sections indicating injection locations. **(A2,B2)** Left: Low-magnification images showing the distribution of M1 and M2 axons (magenta) in the lateral striatum, overlaid with MOR immunostaining (green). The sections at 3.4 mm lateral to the midline are represented. Middle and Right: Higher magnification views of the boxed regions in the left panels. Axonal labeling is shown in the middle panels; MOR staining is shown in the right panels. The scale bars shown in **(A)** also apply to **(B)**. **(C)** Quantification of axonal distribution in matrix and striosome compartments. The ratio of axonal fluorescence intensity in the matrix relative to the striosome is plotted for the striatal sections > 2.9 mm lateral to the midline (*L* > 2.9 mm). A significant difference was observed. ***p < 0.001. **(D)** Projections from M1 and M2 to the ventral thalamus. Calbindin or parvalbumin immunostaining was used to delineate the boundaries of thalamic nuclei. PC, paracentral nucleus; Rt, thalamic reticular nucleus; VA, ventral anterior nucleus; VL, ventrolateral nucleus; VM, ventromedial nucleus.

To this end, biotinylated dextran amine (BDA), an anterograde tracer, was injected into either the M1 or M2 (Figures 5A1,B1). The axonal distribution within striatal compartments was examined using immunofluorescent labeling of MOR (Figures 5A2,B2). The results showed that both M1- and M2-derived axons projected to the dorsolateral striatum, consistent with previous reports, albeit with subtle topographical differences. Notably, M1 axons were densely distributed within the matrix compartment compared to the striosome, whereas M2 axons exhibited a more homogeneous pattern across compartments (Figures 5A2,B2). Quantitative analysis of fluorescence intensity revealed that M1 axons preferentially targeted the matrix over the striosome, whereas M2 axons lacked significant compartmental selectivity. The ratio of axonal fluorescence was calculated as the fluorescent intensity in the matrix divided by that in the adjacent striosome (Figure 5C; *N* = 3 rats). For M1 projections, the ratio was 1.43 ± 0.26 in the lateral striatum (> 2.9 mm lateral to the midline; *N* = 155 ROIs) and 1.37 ± 0.20 in the medial striatum (< 2.9 mm lateral to the midline; *N* = 60 ROIs), indicating no statistically significant difference (*p* = 0.19). For M2 projections, the ratio was 1.35 ± 0.31 (*N* = 106 ROIs) in the lateral striatum and 1.16 ± 0.22 in the medial striatum (*N* = 140 ROIs), indicating a statistically significant difference (*p* = 1.14 × 10^–7^). All ratios exceeded 1.0, the expected value under the null hypothesis (M1 medial, *p* < 2.2 × 10^–16^; M2 medial, *p* = 6.5 × 10^–14^; M1 lateral, *p* < 1.67 × 10^–11^; M2 lateral, *p* < 2.2 × 10^–16^). Comparison between M1 and M2, a significant difference was observed in the lateral striatum (*p* < 2.2 × 10^–16^), whereas no significant difference was detected in the medial striatum (*p* = 0.12). These results suggest that preferential projections to the matrix are more prominent for M1 axons than for M2 axons in the lateral striatum, although both M1 and M2 exhibit a preference to the matrix compartment. In addition, dense axonal projections from both M1 and M2 were observed in the ventral thalamic nuclei (Figures 5D1,D2).

**FIGURE 6:**
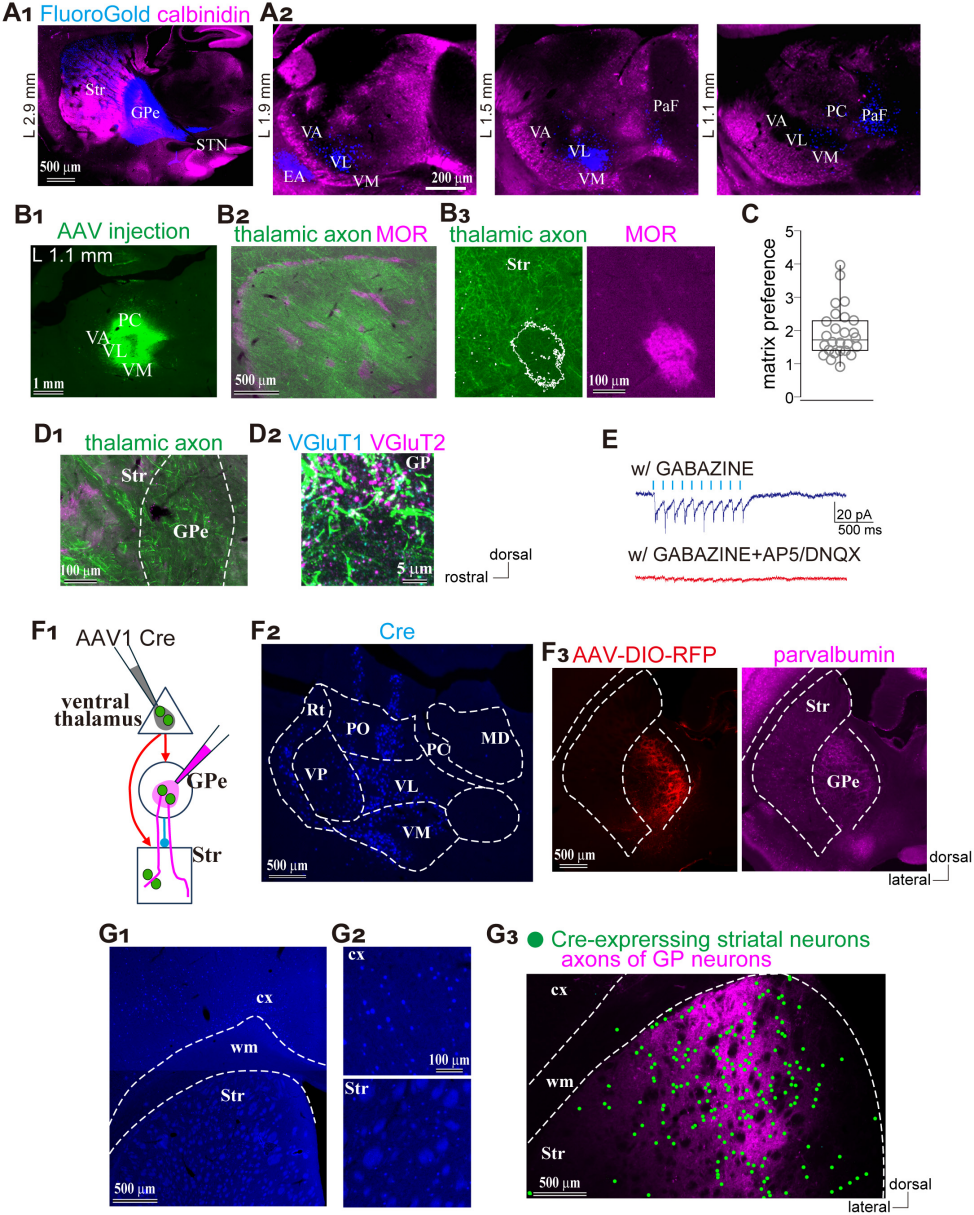
Preferential thalamic projection to the striatal matrix compartment with GPe innervation. **(A)** Distribution of neurons projecting to the GPe. **(A1)** Retrograde tracer (FluoroGold; blue) injection into the GPe. Calbindin immunostaining (magenta) is overlaid to distinguish brain regions. **(A2)** Distribution of the retrogradely labeled neurons (blue) in the thalamus. Three sagittal planes are shown. **(B)** Ventral thalamic nuclei preferentially projected to the striatal matrix compartments. **(B1)** AAV expressing GFP injections into the thalamus. **(B2)** Low-magnification image showing the distribution of AAV-labeled axons in the striatum, overlaid with MOR staining (magenta). **(B3)** Higher magnification view including the matrix and striosome. **(C)** Quantification of the ratio of axonal fluorescent intensity in the matrix relative to the striosome. **(D)** Thalamic axons emitted collaterals in the GPe. **(D1)** Low-magnification image showing thalamic axons in the striatum and GPe. **(D2)** Higher magnification image of the GPe with immunostaining for VGluT1 (cyan) and VGluT2 (magenta). Brain orientation for **(A–D)** is indicated to the right of D2. **(E)** Electrophysiological confirmation of synaptic connections from the ventral thalamic axons to the GPe neurons. Inward currents were elicited by optic stimulation (cyan ticks) in the presence of Gabazine (top trace). Additional application of glutamate receptor antagonists abolished the inward currents (bottom trace). **(F)** Simultaneous visualization of striatal neurons and GPe neurons receiving motor thalamic inputs. **(F1)** Schematic illustration of AAV injections. **(F2)** AAV1-Cre injection in the ventral thalamus, visualized by Cre immunostaining (blue). **(F3)** Visualization of GPe neurons innervated by the thalamus using AAV-DIO-RFP injection (red; left). PV-immunostaining was performed to identify the GPe (magenta; right). **(G)** Spatial overlap between striatal neurons and GPe axons innervated by the same thalamic neuronal population. **(G1,G2)** Cre expressing cortical and striatal neurons visualized by Cre immunostaining (blue). Magnified views are shown in **(G2,G3)** Spatial distribution of labeled striatal neurons (marked by green circles) and GPe axons (magenta). EA, extended amygdala; MD, mediodorsal nucleus; PC, paracentral nucleus; PaF, parafascicular nucleus; PO, posterior nucleus; Rt, thalamic reticular nucleus; Str, striatum; STN, subthalamic nucleus; VA, ventral anterior nucleus; VL, ventrolateral nucleus; VM, ventromedial nucleus; VP, ventral posterior nucleus.

As another potential excitatory source that may drive the feedforward inhibitory pathway, we examined whether the GPe receives thalamic projections using FluoroGold as a retrograde tracer (*N* = 4 rats; Figure 6A1). Retrogradely labeled neurons were observed in the striatum, STN, SN, and thalamus (Figures 6A1,A2). Within the thalamus, labeled neurons were found in the ventral anterior (VA) nuclei, ventrolateral (VL), and ventromedial (VM), as well as in the parafascicular nucleus (PaF) of the intralaminar group (Figure 6A2), consistent with earlier reports on thalamostriatal projections (Smith and Parent, 1986; Cheatwood et al., 2005). Since the VA, VL, and VM collectively comprise the ventral thalamic nuclei—commonly referred to as the motor thalamus—and receive inputs from motor cortical areas (Figure 5D), we focused our subsequent analyses on these regions. To this end, we injected an anterograde AAV vector expressing GFP and ChR2 into the VA/VL/VM complex (*N* = 6 rats; Figures 6B,C), to examine the distribution of thalamic axons. Labeled axons extended from the thalamus and projected densely to both the striatum and cerebral cortex. We quantitatively analyzed their distribution in the striatum using MOR immunostaining (Figures 6B2,B3). The ratio of fluorescent intensity in the matrix to the striosome was 1.94 ± 0.75 (*N* = 3 rats; 26 ROIs per compartment; Figure 6C), indicating a significant deviation from the null hypothesis value of 1, which assumes no compartmental difference (*p* = 5.96 × 10^–8^ Wilcoxon signed-rank test). These findings suggest that ventral thalamic nuclei preferentially target the matrix compartment over the striosome.

One remaining question is whether these thalamic axons innervate the GPe. In the same samples, numerous axons were also observed within the GPe along their trajectory to the striatum and cerebral cortex (Figure 6D1). To determine whether these axons formed synaptic terminals in the GPe, we performed immunostaining for VGluT2. Co-localization of VGluT2-positive puncta with labeled axons indicated the presence of functional glutamatergic thalamic terminals (Figure 6D2). To verify synaptic connectivity onto GPe neurons, we conducted whole-cell patch-clamp recordings. As shown in Figure 6E, light stimulation elicited synaptic currents that were abolished by the application of AP5 and DNQX, confirming that ventral thalamic nuclei directly innervate GPe neurons via glutamatergic synapses. These findings suggest that the ventral thalamic nuclei represent an additional source of excitatory input to the GPe–striatal feedforward inhibitory circuit, potentially contributing to the precise temporal regulation of striatal activity.

Finally, we examined whether GPe neurons innervated by thalamic nuclei project to striatal regions that also receive input from the same thalamic source. To address this, AAV1-hSyn-Cre was injected into the motor thalamic nuclei (VA/VL/VM), followed by a second injection of AAV-Flex-tdTomato into the GPe (*N* = 4 rats; Figure 6F1). Immunohistochemical detection of Cre expression confirmed the injection site in the thalamus and revealed anterograde trans-synaptically labeled neurons in both the striatum and GPe (Figures 6F2,F3). Cre expression was observed in VA/VL/VM at the injection site and in the reticular thalamic nucleus (Rt), which likely receives intra-thalamic projections from these nuclei. Cre expression was also detected in the dorsal striatum and motor cortex, both known targets of motor thalamic projections (Figures 6G1,G2). Moreover, Cre-driven tdTomato expression was confined to the GPe (Figure 6F3) tdTomato-labeled GPe axons were distributed in regions overlapping with Cre-expressing areas of the striatum (Figure 6G3). These observations suggest that striatal neurons innervated by the motor thalamus and axons from GPe neurons receiving input from the same thalamic source converge within the same striatal space, forming a putative feedforward circuit.

## 4 Discussion

**FIGURE 7:**
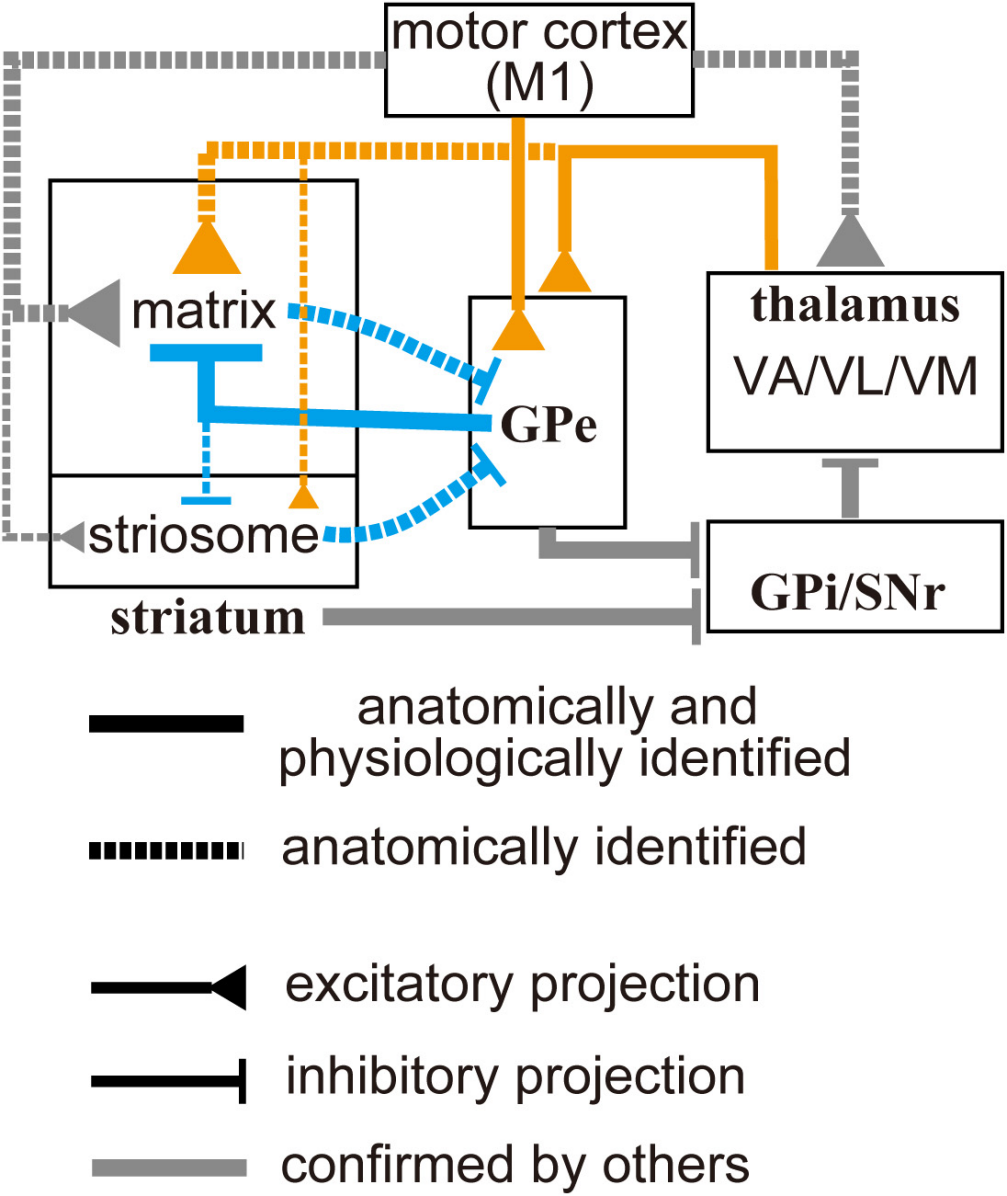
Schematic diagram of the motor-loop within the cortico-basal ganglia-thalamic circuit. Gray lines indicate connections previously identified in other studies (see text for details). Orange and blue lines represent connections observed in the present study: solid lines indicate projections confirmed both anatomically and electrophysiologically; dotted lines indicate projections identified only anatomically. Line thickness reflects the relative strength of each projection. Cell types are omitted from the diagram to avoid excessive complexity. Ventral thalamic nuclei, as well as the primary motor cortex, selectively project to the striatal matrix compartment. In addition, they can also drive the globus pallidus external segment, which can provide feedforward inhibition to the matrix. This topographically organized excitation/inhibition may contribute to spatially and temporally selective activation of specific striatal neuron populations.

In this study, we demonstrated that both pallidosubthalamic and pallidostriatal GPe neurons preferentially project to the matrix compartment of the striatum and robustly innervate CINs. In addition, motor thalamic nuclei exhibit a similar bias toward the matrix compartment and provide excitatory input to GPe neurons. These findings suggest that the matrix compartment may serve as a key node for integrating excitatory and inhibitory signals within the basal ganglia circuitry (see Figure 7). In the following sections, we discuss the implications of these findings for striatal function and basal ganglia organization. It should be noted, however, that our conclusions are based on anatomical tracing and *ex vivo* electrophysiological recordings, which reflect static circuit architecture rather than dynamic or behaviorally relevant activity. As such, the functional roles of these matrix-selective connections involving the GPe remain hypothetical and warrant further investigation *in vivo*.

### 4.1 Selective inhibitory projections to the striatal compartments: matrix bias and implications

AAV-assisted tracing revealed a matrix-preferential projection pattern originating from the GPe. Immunohistochemical labeling indicated that pallidosubthalamic neurons were predominantly Nkx2.1-positive, with only few FoxP2-positive cells, consistent with a prototypic neuronal identity (Abdi et al., 2015; Hernandez et al., 2015). The pallidostriatal population included both FoxP2-positive and -negative neurons, with a slight predominance of the former. Although axonal density in the striatum was higher in the pallidostriatal labeling, both tracing approaches consistently demonstrated a matrix bias, suggesting that matrix preference is a general property of GPe neurons regardless of cell type. Smith et al. (2016) reported no significant difference in the proportion of rabies-labeled GPe neurons between the striosome and matrix compartments. This discrepancy may be explained by the high sensitivity of rabies virus labeling, which can label input neurons equally regardless of axon terminal density or synaptic strength. Thus, rabies-based input mapping may not directly reflect the functional weight of synaptic connections.

In our study, the combination of AAV1-Flp injection into the GPe and AAV-fDIO-GFP injection into the striatum resulted in strongly biased labeling within the matrix compartment (Figure 4A). In contrast, retrograde tracing using FluoroGold suggested that both striosome and matrix compartments project comparably to the GPe. These findings indicate that AAV1-Flp primarily labeled neurons anterogradely, with minimal retrograde labeling (Zingg et al., 2017, 2020; Karube et al., 2024). Taken together, these results suggest that information from the striatum can converge at the GPe regardless of compartmental or cell-type origin. This convergence may facilitate the integration of cross-compartmental signals and reflect non-motor functions of the GPe (Aristieta and Gittis, 2021; Courtney and Chan, 2023). However, since our analysis was limited to neuronal counts, potential differences in synaptic strength may still exist in the striatal compartment outputs to the GPe.

Furthermore, our study revealed potential heterogeneity in VGAT expression within the striosome compartment (Figure 1F). A subset of striosome islands exhibited weak VGAT expression, which correlated with sparse GPe inputs. In contrast, other striosome islands with few GPe inputs showed VGAT expression levels comparable to those in the surrounding matrix. These findings suggest that GABAergic inhibition may vary among subregions of the striosome. Indeed, the chemical composition of the striosome is highly diverse (Miyamoto et al., 2018), and the associated circuitry may differ accordingly. The mechanisms and heterogeneity of GABAergic inhibition in the striosome remain open questions.

### 4.2 Compartment-selective excitatory inputs: cortical and thalamic pathways

Fujiyama et al. (2006) demonstrated a homogeneous distribution of VGluT1 across the striatum, whereas VGluT2 expression was strongly biased toward the matrix compartment. In the present study, we showed that both M1 and M2 preferentially innervate the matrix compartment of the lateral striatum, with M1 exhibiting a stronger bias (Figure 5). In the medial striatum, M2 axons projected comparably to the matrix and striosome compartments, suggesting a higher-order functional role for M2. These differences in matrix preference, with respect to cortical origins and striatal dimensions, may reflect functional specializations across striatal regions.

Axonal tracing studies have identified thalamic nuclei as sources of matrix-selective inputs, with specificity depending on their origin. The PaF is a representative nucleus that selectively innervates the matrix compartment (Crittenden and Graybiel, 2011; Smith et al., 2014; Fujiyama et al., 2019). Our tracing data demonstrate that motor thalamic nuclei— including VA, VL, and VM—also preferentially target the matrix compartment. Notably, VA, VL, and VM have been shown to form functional synaptic contacts with GPe neurons (Figure 6), as suggested by rabies viral tracing (Cui et al., 2021a). The ventral thalamic nuclei are interconnected with the motor cortex, forming part of the motor loop within the cortico-basal ganglia-thalamic circuit. In addition, both cerebellar excitatory inputs and GABAergic projections from the basal ganglia innervate the VA/VL complex, forming two distinct subzones in rodents (Kuramoto et al., 2009). Our data did not distinguish the cellular origin of thalamopallidal inputs—whether they convey basal ganglia or cerebellar outputs—highlighting the need for further studies to clarify their functional implications.

Given the matrix-selective projections from the motor cortical areas, motor thalamic nuclei, and the GPe, these excitatory and inhibitory innervations may be functionally counterbalanced. Moreover, the GPe itself receives excitatory input from both the cortex and thalamus, suggesting that pallidostriatal projections may act as a feedforward mechanism to terminate excitatory states in striatal neurons. Although our study revealed macro-scale convergence rather than single-cell resolution, this circuitry may represent a mechanism for fine-tuning striatal activity.

### 4.3 GPe innervation to CINs and MSNs

Our data demonstrate that pallidostriatal IPSCs are larger in CINs than in MSNs within the matrix compartment (Figures 3D,E), suggesting that GPe neurons exert stronger inhibitory control over CINs. In addition, when using a CsCl-based electrode solution, which improves space-clamp quality, both the connection probability and amplitude of IPSCs increased in MSNs, whereas those in CINs remained largely unchanged. This suggests that GPe synapses onto CINs are likely located at proximal portions of individual neurons, whereas those onto MSNs are more distally positioned. Indeed, morphological data strongly support this interpretation, showing frequent somatic and proximal dendritic appositions onto CINs (Figure 3F). These features imply functionally potent inhibition from the GPe to CINs, likely exerting global control over the integrate activity of individual CINs through proximal synaptic targeting. Anatomical observations in rats have demonstrated that arkypallidal neurons innervate striatal interneurons, including CINs, as well as MSNs (Mallet et al., 2012). Basket-like terminals surrounding interneuron somata were observed, consistent with our findings of strong GPe innervation of CINs. The present result is also consistent with previous findings showing that GPe inputs to MSNs predominantly target distal dendrites (Glajch et al., 2016). Electron microscopic observations have further revealed that arkypallidal neurons frequently form synapses on dendrites and dendritic spines (Mallet et al., 2012). Taken together, these structural features support the notion that proximally located GABAergic synapses from the GPe to CINs mediate strong inhibitory control. Our IPSC recordings revealed relatively large variability in IPSC amplitude (Figure 3F). Together with the observation that GPe input was not detected in a subset of CINs, this suggests that GPe innervation may differentiate among CINs, potentially reflecting the heterogeneous nature (Ahmed et al., 2019).

Previous studies have reported GPe cell type-dependent differences in pallidostriatal connections, particularly between Npas1-positive (including arkypallidal) and PV-positive (prototypic) GPe neurons. Npas1-positive neurons elicit larger IPSCs in iMSNs than in dMSNs, whereas PV-positive prototypic neurons induce weaker IPSCs in both subtypes (Glajch et al., 2016; Cui et al., 2021a), although their relationship to striatal compartments was not addressed. In our study, pallidostriatal neurons labeled by a combination of AAVs included approximately 55% arkypallidal neurons (Figure 2B), indicating that a substantial portion of prototypic neurons also give rise to pallidostriatal axons (Abdi et al., 2015; Fujiyama et al., 2016; Mizutani et al., 2017). Although synaptic influence may be stronger in arkypallidal neurons due to their extremely dense axonal arborization in the striatum (Figures 2C,D; Mallet et al., 2012; Fujiyama et al., 2016), we cannot exclude the possibility of differential synapse formation between prototypic and arkypallidal neurons in their innervation of CINs. In addition, this may account for the relatively lower connection probability from the GPe to MSNs observed in our study compared to previous studies in mice that specifically targeted Npas1-positive neurons.

### 4.4 CINs in the context of cortical and thalamic inputs

Striatal CINs receive convergent excitatory inputs from both cortical and thalamic sources and are thought to modulate striatal circuitry through integrative mechanisms. In this section, we explore how these afferent pathways may interact with GPe-mediated inhibition. Previous studies in mice have uncovered neural connections between the striatum and GPe, particularly through cell type–specific labeling approaches. It should be noted, however, that our present results are not derived from purely cell type–selective labeling, and therefore some discrepancies or unresolved differences may remain, particularly when compared to studies in mice using genetically defined populations. Thalamic synapses onto CINs tend to target proximal dendrites and evoke burst–pause firing patterns, which are modulated by dopaminergic tone. In contrast, cortical inputs typically target distal dendrites and elicit smaller postsynaptic responses in CINs compared to those in MSNs or PV-positive interneurons (Johansson and Silberberg, 2020), which may lead to simple phasic activation (Ding et al., 2008; Assous, 2021; Ratna and Francis, 2025). The burst–pause firing of CINs has been proposed to gate cortico-striatal excitation via cholinergic modulation, thereby promoting selective activation of iMSNs that facilitate NoGo responses (Ding et al., 2010). A recent study in mice reported that iMSNs selectively suppress prototypic GPe neurons, which can lead to disinhibition of arkypallidal neurons (Aristieta et al., 2021; Jones et al., 2023). This disinhibition could enhance GPe-mediated inhibition of CINs in the striatum. This, in turn, may modulate acetylcholine release and influence local striatal circuitry (Abudukeyoumu et al., 2019; Assous, 2021), including the regulation of glutamatergic synapses and dopamine release machinery (Gonzales and Smith, 2015; Assous, 2021). Contrary to the preceding assumption, *in vivo* recordings have demonstrated that suppression of prototypic neurons does not reliably induce disinhibition of arkypallidal neurons (Johansson and Ketzef, 2023), suggesting that the functional impact of this interaction may depend on behavioral state or network context. Further studies are required to elucidate the physiological and behavioral impact of this pathway. In contrast, cortical activation of CINs is relatively weak (Johansson and Silberberg, 2020). Under conditions dominated by cortical activation, pallidostriatal inhibition may play a distinct role in shaping striatal circuitry compared to states dominated by thalamic input. Given the reciprocal excitatory connections between cortex and thalamus, thalamus-driven and cortex-driven states may interact dynamically *in vivo*, and the resulting behavior of the striatal circuit is likely to be more complex than either pathway alone would suggest.

While previous studies on thalamus-related basal ganglia circuitry have focused primarily on the intralaminar nuclei (Smith et al., 2014), it remains unclear whether similar principles apply to the ventral motor thalamus. Unlike the intralaminar nuclei, which can be driven by external salient signals independently of cortical inputs, the motor thalamus is unlikely to be activated in isolation due to its strong cerebral or cerebellar afferents. Thus, cortical and the thalamic excitation may act in concert. These considerations raise the possibility that CINs associated with the motor cortex and thalamus may integrate motor-related signals and influence activity in the cortex, striatum, and GPe. In this context, CINs may act heterogeneously depending on external input sources. This heterogeneity may contribute to differential local and long-range circuit integration, potentially shaping striatal output in a context-dependent manner. These considerations highlight the integrative potential of CINs within striatal microcircuits. In the following section, we expand our focus to the GPe itself, examining its role as a central hub coordinating activity across multiple basal ganglia structures.

### 4.5 Functional implications of GPe-centered matrix circuitry: toward a revised basal ganglia model

Recent advances in basal ganglia research have led to a revised framework in which the GPe is regarded as a central hub, interconnecting with multiple nuclei both within and beyond the basal ganglia (Hegeman et al., 2016; Courtney and Chan, 2023; Fang and Creed, 2024). Consistent with this emerging perspective, our findings suggest that the GPe plays a pivotal role in modulating striatal activity through the integration of cortical and thalamic inputs.

Intriguingly, the matrix compartment appears to selectively receive extra-striatal GABAergic inhibition from the GPe, despite the presence of local GABAergic interneurons in both the matrix and striosome compartments. These local interneurons may receive inputs similar to those of MSNs and contribute to feedforward inhibition within local striatal microcircuits (Tepper et al., 2010, 2018; Silberberg and Bolam, 2015). As demonstrated in our study and previous reports, the striatum and GPe likely share excitatory inputs from both the cerebral cortex and thalamus. In this context, intra-striatal and extra-striatal inhibitory mechanisms may operate in parallel. A key distinction arises from the striatopallidal projections, which are thought to originate from both the matrix and striosome compartments (Figures 4B,D; Smith et al., 2016). One unresolved issue for future studies is the potential differential contribution of dMSNs and iMSNs, given that the proportion of axonal varicosities in the GPe differs significantly between these two cell types (Fujiyama et al., 2011), and that their distribution across the matrix and striosome compartments also varies (Crittenden and Graybiel, 2011). Thus, information from prefrontal and limbic cortical areas to the striosome, and from motor and sensory areas targeting the matrix, may converge at the level of the GPe. Through this convergence, the matrix compartment could indirectly access striosomal activity via GPe-mediated inhibition, although cross-compartmental integration may also occur locally via interneurons, independent of GPe inputs. Integration of information across distinct cortical layers and cell types may be further mediated by pallidostriatal and thalamostriatal pathways. The matrix compartment predominantly receives inputs from upper cortical layers, which mainly contain intra-telencephalic (IT) neurons, whereas the striosome is primarily innervated by deep layers, including pyramidal tract (PT) neurons that project to deep subcortical structures such as the brainstem and spinal cord (Wilson, 1987; Gerfen, 1989; Shipp, 2017). Moreover, both the thalamus and GPe receive axon collaterals exclusively from PT neurons, but not from IT neurons (Karube et al., 2019; Abecassis et al., 2020). Therefore, thalamostriatal and pallidostriatal projections may convey PT-related signals that are potentially involved in motor output commands. Recently, Koster and Sherman (2024) revealed that motor cortical inputs and GPi/SNr inputs converge onto single neurons within the VM/VL complex. This convergence suggests that the motor thalamus may serve as an integrating node for these projections, consistent with our observation of spatial overlap between thalamostriatal and thalamo-pallido-striatal projections. Our current results showing projections from VL/VM to the GPe may contribute to modulating this circuitry via pallidostriatal–striatofugal and pallidofugal pathways. In this context, cell type–dependent contributions within the GPe and striatum should be further examined in future studies. Although anatomical studies have shown that both PT and IT neurons project to the same striatal compartments (Levesque and Parent, 1998; Smith et al., 2016), the synaptic or functional contributions of each pathway remain incompletely understood. Taken together, pallidostriatal projections may serve to integrate motor-related signals derived from multiple sources.

While we emphasize excitatory cortical and thalamic activation of GPe neurons based on the current results, other excitatory and inhibitory sources also contribute to GPe activity. One important pathway involves the STN, which provides dense excitatory input to the GPe but only sparse innervations of the striatum. The STN receives cortical input via the hyperdirect pathway from layer 5 pyramidal neurons, which also send axon collaterals to both the striatum and the GPe (Kita and Kita, 2012). Given the differential cortical layer inputs to the striosome and matrix described above, it is plausible that signals destined for deep subcortical structures converge at the GPe via striatopallidal, subthalamopallidal, and corticopallidal pathways. In addition, arkypallidal neuron activity is modulated by intra-GPe inhibition from prototypic neurons, as well as cell type–dependent inhibition from MSNs (Aristieta et al., 2021; Ketzef and Silberberg, 2021). Previous studies in mice have distinguished D1- and D2-type MSNs in their projections to the GPe, whereas our study focuses on their compartmental origin (matrix vs. striosome). These perspectives may be complementary, and future studies should aim to resolve their intersection. Collectively, these observations suggest that GPe-mediated inhibition of the striatum may convey information not directly transmitted to the striatum itself, thereby enabling indirect cross-compartmental and cross-regional communication and integration.

Recent studies have identified heterogeneous neuronal populations in the primate GPe, suggesting functional diversity beyond the classical indirect pathway (Yoshida and Hikosaka, 2025; Katabi et al., 2023). Although the presence of arkypallidal neurons in primates remains unconfirmed, axon collaterals from the GPe to the striatum have been reported (Sato et al., 2000). These physiological and anatomical features imply that recent advances in basal ganglia circuitry may be applicable to primates, including humans. Our findings in rodents may therefore provide a framework for re-evaluating GPe-centered circuits in higher species. GPe connectivity and activity are altered in rodent models of Parkinson’s disease (Glajch et al., 2016; Cui et al., 2021a; Dong et al., 2021). Selective modulation of distinct GPe neuron subtypes has been shown to mitigate pathological impairments (Mastro et al., 2017; Spix et al., 2021). Building on this, computational models incorporating detailed cell types and connectivity have suggested that cell type– and pathway–dependent mechanisms may contribute to altered neural activity, including the generation of pathological oscillations and phase transitions (Chakravarty et al., 2022; Gast et al., 2021). These insights may help clarify how GPe dysfunction contributes to basal ganglia pathophysiology and inform future therapeutic strategies.

## Data Availability

The original contributions presented in this study are included in this article/supplementary material, further inquiries can be directed to the corresponding authors.

## Footnotes

1
http://www.neuromatic.thinkrandom.com

2
http://www.r-project.org/

## References

[B1] Abdi A. Mallet N. Mohamed F. Y. Sharott A. Dodson P. D. Nakamura K. C. (2015). Prototypic and arkypallidal neurons in the dopamine-intact external globus pallidus. *J. Neurosci.* 35 6667–6688. 10.1523/JNEUROSCI.4662-14.2015 25926446 PMC4412890

[B2] Abecassis Z. A. Berceau B. L. Win P. H. Garcia D. Xenias H. S. Cui Q. (2020). Npas1(+)-Nkx2.1(+) neurons are an integral part of the cortico-pallido-cortical Loop. *J. Neurosci.* 40 743–768. 10.1523/JNEUROSCI.1199-19.2019 31811030 PMC6975296

[B3] Abudukeyoumu N. Hernandez-Flores T. Garcia-Munoz M. Arbuthnott G. W. (2019). Cholinergic modulation of striatal microcircuits. *Eur. J. Neurosci.* 49 604–622. 10.1111/ejn.13949 29797362 PMC6587740

[B4] Ahmed N. Y. Knowles R. Dehorter N. (2019). New insights into cholinergic neuron diversity. *Front. Mol. Neurosci.* 12:204. 10.3389/fnmol.2019.00204 31551706 PMC6736589

[B5] Albin R. L. Young A. B. Penney J. B. (1989). The functional anatomy of basal ganglia disorders. *Trends Neurosci.* 12 366–375. 10.1016/0166-2236(89)90074-x 2479133

[B6] Alexander G. E. Crutcher M. D. (1990). Functional architecture of basal ganglia circuits: Neural substrates of parallel processing. *Trends Neurosci.* 13 266–271. 10.1016/0166-2236(90)90107-l 1695401

[B7] Arber S. Costa R. M. (2022). Networking brainstem and basal ganglia circuits for movement. *Nat. Rev. Neurosci.* 23 342–360. 10.1038/s41583-022-00581-w 35422525

[B8] Aristieta A. Gittis A. (2021). Distinct globus pallidus circuits regulate motor and cognitive functions. *Trends Neurosci.* 44 597–599. 10.1016/j.tins.2021.06.001 34144845 PMC8562495

[B9] Aristieta A. Barresi M. Azizpour Lindi S. Barrière G. Courtand G. de la Crompe B. (2021). A Disynaptic circuit in the globus pallidus controls locomotion inhibition. *Curr. Biol.* 31 707–721.e7. 10.1016/j.cub.2020.11.019 33306949

[B10] Assous M. (2021). Striatal cholinergic transmission. Focus on nicotinic receptors’ influence in striatal circuits. *Eur. J. Neurosci.* 53 2421–2442. 10.1111/ejn.15135 33529401 PMC8161166

[B11] Bernácer J. Prensa L. Giménez-Amaya J. M. (2007). Cholinergic interneurons are differentially distributed in the human striatum. *PLoS One* 2:e1174. 10.1371/journal.pone.0001174 18080007 PMC2137841

[B12] Bernácer J. Prensa L. Giménez-Amaya J. M. (2012). Distribution of GABAergic interneurons and dopaminergic cells in the functional territories of the human striatum. *PLoS One* 7:e30504. 10.1371/journal.pone.0030504 22272358 PMC3260284

[B13] Bevan M. D. Booth P. A. Eaton S. A. Bolam J. P. (1998). Selective innervation of neostriatal interneurons by a subclass of neuron in the globus pallidus of the rat. *J. Neurosci.* 18 9438–9452. 10.1523/JNEUROSCI.18-22-09438.1998 9801382 PMC6792890

[B14] Bogacz R. Moraud E. M. Abdi A. Magill P. J. Baufreton J. (2016). Properties of neurons in external globus pallidus can support optimal action selection. *PLoS Comput. Biol.* 12:e1005004. 10.1371/journal.pcbi.1005004 27389780 PMC4936724

[B15] Chakravarty K. Roy S. Sinha A. Nambu A. Chiken S. Kotaleski J. H. (2022). Transient response of basal ganglia network in healthy and low-dopamine state. *eNeuro* 9:0376-21. 10.1523/ENEURO.0376-21.2022 35140075 PMC8938981

[B16] Cheatwood J. L. Corwin J. V. Reep R. L. (2005). Overlap and interdigitation of cortical and thalamic afferents to dorsocentral striatum in the rat. *Brain Res.* 1036 90–100. 10.1016/j.brainres.2004.12.049 15725405

[B17] Corbit V. L. Whalen T. C. Zitelli K. T. Crilly S. Y. Rubin J. E. Gittis A. H. (2016). Pallidostriatal projections promote beta oscillations in a dopamine-depleted biophysical network model. *J. Neurosci.* 36 5556–5571. 10.1523/JNEUROSCI.0339-16.2016 27194335 PMC4871989

[B18] Courtney C. D. Chan C. S. (2023). Cell type-specific processing of non-motor signals in the external pallidum. *Trends Neurosci.* 46 336–337. 10.1016/j.tins.2023.03.002 36935263 PMC11107425

[B19] Courtney C. D. Pamukcu A. Chan C. S. (2023). Cell and circuit complexity of the external globus pallidus. *Nat .Neurosci.* 26 1147–1159. 10.1038/s41593-023-01368-7 37336974 PMC11382492

[B20] Cowan R. L. Wilson C. J. Emson P. C. Heizmann C. W. (1990). Parvalbumin-containing gabaergic interneurons in the rat neostriatum. *J. Comp. Neurol.* 302 197–205. 10.1002/cne.903020202 2289971

[B21] Crittenden J. R. Graybiel A. M. (2011). Basal ganglia disorders associated with imbalances in the striatal striosome and matrix compartments. *Front. Neuroanat.* 5:59. 10.3389/fnana.2011.00059 21941467 PMC3171104

[B22] Crittenden J. R. Tillberg P. W. Riad M. H. Shima Y. Gerfen C. R. Curry J. (2016). Striosome-dendron bouquets highlight a unique striatonigral circuit targeting dopamine-containing neurons. *Proc. Natl. Acad. Sci. U S A.* 113 11318–11323. 10.1073/pnas.1613337113 27647894 PMC5056098

[B23] Cui Q. Du X. Chang I. Y. M. Pamukcu A. Lilascharoen V. Berceau B. L. (2021a). Striatal direct pathway targets Npas1+pallidal neurons. *J. Neurosci.* 41 3966–3987. 10.1523/JNEUROSCI.2306-20.2021 33731445 PMC8176753

[B24] Cui Q. Pamukcu A. Cherian S. Chang I. Y. M. Berceau B. L. Xenias H. S. (2021b). Dissociable roles of pallidal neuron subtypes in regulating motor patterns. *J. Neurosci*. 41 4036–4059. 10.1523/JNEUROSCI.2210-20.2021 33731450 PMC8176746

[B25] Ding J. B. Guzman J. N. Peterson J. D. Goldberg J. A. Surmeier D. J. (2010). Thalamic gating of corticostriatal signaling by cholinergic interneurons. *Neuron* 67 294–307. 10.1016/j.neuron.2010.06.017 20670836 PMC4085694

[B26] Ding J. Peterson J. D. Surmeier D. J. (2008). Corticostriatal and thalamostriatal synapses have distinctive properties. *J. Neurosci.* 28 6483–6492. 10.1523/JNEUROSCI.0435-08.2008 18562619 PMC3461269

[B27] Dodson P. D. Larvin J. T. Duffell J. M. Garas F. N. Doig N. M. Kessaris N. (2015). Distinct developmental origins manifest in the specialized encoding of movement by adult neurons of the external globus pallidus. *Neuron* 86, 501–513. 10.1016/j.neuron.2015.03.007 25843402 PMC4416107

[B28] Dong J. Hawes S. Wu J. Le W. Cai H. (2021). Connectivity and functionality of the globus pallidus externa under normal conditions and Parkinson’s disease. *Front. Neural Circuits* 15:645287. 10.3389/fncir.2021.645287 33737869 PMC7960779

[B29] Fang L. Z. Creed M. C. (2024). Updating the striatal–pallidal wiring diagram. *Nat. Neurosci.* 27 15–27. 10.1038/s41593-023-01518-x 38057614 PMC11892008

[B30] Fenno L. E. Mattis J. Ramakrishnan C. Hyun M. Lee S. Y. He M. (2014). Targeting cells with single vectors using multiple-feature Boolean logic. *Nat. Methods* 11 763–772. 10.1038/nmeth.2996 24908100 PMC4085277

[B31] Flaherty A. W. Graybiel A. M. (1995). Motor and somatosensory corticostriatal projection magnifications in the squirrel monkey. *J. Neurophysiol.* 74 2638–2648. 10.1152/jn.1995.74.6.2638 8747221

[B32] Friend D. M. Kravitz A. V. (2014). Working together: Basal ganglia pathways in action selection. *Trends Neurosci.* 37 301–303. 10.1016/j.tins.2014.04.004 24816402 PMC4041812

[B33] Fujiyama F. Nakano T. Matsuda W. Furuta T. Udagawa J. Kaneko T. (2016). A single-neuron tracing study of arkypallidal and prototypic neurons in healthy rats. *Brain Struct. Funct.* 221 4733–4740. 10.1007/s00429-015-1152-2 26642797

[B34] Fujiyama F. Sohn J. Nakano T. Furuta T. Nakamura K. C. Matsuda W. (2011). Exclusive and common targets of neostriatofugal projections of rat striosome neurons: A single neuron-tracing study using a viral vector. *Eur. J. Neurosci.* 33 668–677. 10.1111/j.1460-9568.2010.07564.x 21314848

[B35] Fujiyama F. Unzai T. Karube F. (2019). Thalamostriatal projections and striosome-matrix compartments. *Neurochem. Int.* 125 67–73. 10.1016/j.neuint.2019.01.024 30710558

[B36] Fujiyama F. Unzai T. Nakamura K. Nomura S. Kaneko T. (2006). Difference in organization of corticostriatal and thalamostriatal synapses between patch and matrix compartments of rat neostriatum. *Eur. J. Neurosci.* 24 2813–2824. 10.1111/j.1460-9568.2006.05177.x 17156206

[B37] Gast R. Gong R. Schmidt H. Meijer H. G. E. Knösche T. R. (2021). On the role of arkypallidal and prototypical neurons for phase transitions in the external pallidum. *J. Neurosci.* 41 6673–6683. 10.1523/JNEUROSCI.0094-21.2021 34193559 PMC8336705

[B38] Gerfen C. R. (1984). The neostriatal mosaic: Compartmentalization of corticostriatal input and striatonigral output systems. *Nature* 311 461–464. 10.1038/311461a0 6207434

[B39] Gerfen C. R. (1985). The neostriatal mosaic. I. Compartmental organization of projections from the striatum to the substantia nigra in the rat. *J. Comp. Neurol.* 236 454–476. 10.1002/cne.902360404 2414339

[B40] Gerfen C. R. (1989). The neostriatal mosaic: Striatal patch-matrix organization is related to cortical lamination. *Science* 246 385–388. 10.1126/science.2799392 2799392

[B41] Giossi C. Rubin J. E. Gittis A. Verstynen T. Vich C. (2024). Rethinking the external globus pallidus and information flow in cortico-basal ganglia-thalamic circuits. *Eur. J. Neurosci.* 60 6129–6144. 10.1111/ejn.16348 38659055

[B42] Glajch K. E. Kelver D. A. Hegeman D. J. Cui Q. Xenias H. S. Augustine E. C. (2016). Npas1+ pallidal neurons target striatal projection neurons. *J. Neurosci.* 36 5472–5488. 10.1523/JNEUROSCI.1720-15.2016 27194328 PMC4871984

[B43] Goenner L. Maith O. Koulouri I. Baladron J. Hamker F. H. (2021). A spiking model of basal ganglia dynamics in stopping behavior supported by arkypallidal neurons. *Eur. J. Neurosci.* 53 2296–2321. 10.1111/ejn.15082 33316152

[B44] Gonzales K. K. Smith Y. (2015). Cholinergic interneurons in the dorsal and ventral striatum: Anatomical and functional considerations in normal and diseased conditions. *Ann. N. Y. Acad. Sci.* 1349 1–45. 10.1111/nyas.12762 25876458 PMC4564338

[B45] Hegeman D. J. Hong E. S. Hernandez V. M. Chan C. S. (2016). The external globus pallidus: Progress and perspectives. *Eur. J. Neurosci.* 43 1239–1265. 10.1111/ejn.13196 26841063 PMC4874844

[B46] Herkenham M. Pert C. B. (1981). Mosaic distribution of opiate receptors, parafascicular projections and acetylcholinesterase in rat striatum. *Nature* 291 415–418. 10.1038/291415a0 6165892

[B47] Hernandez V. M. Hegeman D. J. Cui Q. Kelver D. A. Fiske M. P. Glajch K. E. (2015). Parvalbumin+ neurons and Npas1+ neurons are distinct neuron classes in the mouse external globus pallidus. *J. Neurosci.* 35 11830–11847. 10.1523/JNEUROSCI.4672-14.2015 26311767 PMC4549397

[B48] Holt D. J. Graybiel A. M. Saper C. B. (1997). Neurochemical architecture of the human striatum. *J. Comp. Neurol.* 384 1–25. 10.1002/(SICI)1096-9861(19970721)384:1<1::AID-CNE1>3.0.CO;2-5 9214537

[B49] Ito H. Goto S. Sakamoto S. Hirano A. (1992). Calbindin-D _28*K*_ in the basal ganglia of patients with parkinsonism. *Ann. Neurol.* 32 543–550. 10.1002/ana.410320410 1456738

[B50] Jakab R. L. Hazrati L.-N. Goldman-Rakic P. (1996). Distribution and neurochemical character of substance P receptor (SPR)-immunoreactive striatal neurons of the macaque monkey: Accumulation of SP fibers and SPR neurons and dendrites in ?striocapsules? encircling striosomes. *J. Comp. Neurol.* 369 137–149. 10.1002/(SICI)1096-9861(19960520)369:1<137::AID-CNE10>3.0.CO;2-O 8723708

[B51] Johansson Y. Ketzef M. (2023). Sensory processing in external globus pallidus neurons. *Cell Rep.* 42:111952. 10.1016/j.celrep.2022.111952 36640317

[B52] Johansson Y. Silberberg G. (2020). The functional organization of cortical and thalamic inputs onto five types of striatal neurons is determined by source and target cell identities. *Cell Rep.* 30 1178–1194.e3. 10.1016/j.celrep.2019.12.095 31995757 PMC6990404

[B53] Jones J. A. Higgs M. H. Olivares E. Peña J. Wilson C. J. (2023). Spontaneous activity of the local gabaergic synaptic network causes irregular neuronal firing in the external globus pallidus. *J. Neurosci.* 43 1281–1297. 10.1523/JNEUROSCI.1969-22.2023 36623877 PMC9987574

[B54] Karube F. Takahashi S. Kobayashi K. Fujiyama F. (2019). Motor cortex can directly drive the globus pallidus neurons in a projection neuron type-dependent manner in the rat. *eLife* 8:e49511. 10.7554/eLife.49511 31711567 PMC6863630

[B55] Karube F. Yang Y. Kobayashi K. Fujiyama F. (2024). Anterograde trans-neuronal labeling of striatal interneurons in relation to dopamine neurons in the substantia nigra pars compacta. *Front. Neuroanat.* 18:1325368. 10.3389/fnana.2024.1325368 38482378 PMC10933013

[B56] Katabi S. Adler A. Deffains M. Bergman H. (2023). Dichotomous activity and function of neurons with low- and high-frequency discharge in the external globus pallidus of non-human primates. *Cell Rep.* 42:111898. 10.1016/j.celrep.2022.111898 36596302

[B57] Kawaguchi Y. (1992). Large aspiny cells in the matrix of the rat neostriatum in vitro: Physiological identification, relation to the compartments and excitatory postsynaptic currents. *J. Neurophysiol.* 67 1669–1682. 10.1152/jn.1992.67.6.1669 1352806

[B58] Kawaguchi Y. Wilson C. J. Emson P. C. (1989). Intracellular recording of identified neostriatal patch and matrix spiny cells in a slice preparation preserving cortical inputs. *J. Neurophysiol.* 62 1052–1068. 10.1152/jn.1989.62.5.1052 2585039

[B59] Ketzef M. Silberberg G. (2021). Differential synaptic input to external globus pallidus neuronal subpopulations in vivo. *Neuron* 109 516–529.e4. 10.1016/j.neuron.2020.11.006 33248017

[B60] Kincaid A. E. Wilson C. J. (1996). Corticostriatal innervation of the patch and matrix in the rat neostriatum. *J. Comp. Neurol.* 374 578–592. 10.1002/(SICI)1096-9861(19961028)374:4 8910736

[B61] Kita T. Kita H. (2012). The subthalamic nucleus is one of multiple innervation sites for long-range corticofugal axons: A single-axon tracing study in the rat. *J. Neurosci.* 32 5990–5999. 10.1523/JNEUROSCI.5717-11.2012 22539859 PMC3479642

[B62] Klug J. R. Engelhardt M. D. Cadman C. N. Li H. Smith J. B. Ayala S. (2018). Differential inputs to striatal cholinergic and parvalbumin interneurons imply functional distinctions. *eLife* 7:35657. 10.7554/eLife.35657 29714166 PMC5929909

[B63] Koster K. P. Sherman S. M. (2024). Convergence of inputs from the basal ganglia with layer 5 of motor cortex and cerebellum in mouse motor thalamus. *eLife* 13:e97489. 10.7554/eLife.97489 38856045 PMC11208046

[B64] Kubota Y. Kawaguchi Y. (1993). Spatial distributions of chemically identified intrinsic neurons in relation to patch and matrix compartments of rat neostriatum. *J. Comp. Neurol.* 332 499–513. 10.1002/cne.903320409 8349845

[B65] Kuramoto E. Furuta T. Nakamura K. C. Unzai T. Hioki H. Kaneko T. (2009). Two types of thalamocortical projections from the motor thalamic nuclei of the rat: A single neuron-tracing study using viral vectors. *Cereb. Cortex* 19 2065–2077. 10.1093/cercor/bhn231 19174446

[B66] Labouesse M. A. Torres-Herraez A. Chohan M. O. Villarin J. M. Greenwald J. Sun X. (2023). A non-canonical striatopallidal Go pathway that supports motor control. *Nat. Commun.* 14:6712. 10.1038/s41467-023-42288-1 37872145 PMC10593790

[B67] Levesque M. Parent A. (1998). Axonal arborization of corticostriatal and corticothalamic fibers arising from prelimbic cortex in the rat. *Cereb. Cortex* 8 602–613. 10.1093/cercor/8.7.602 9823481

[B68] Levesque M. Parent A. (2005). The striatofugal fiber system in primates: A reevaluation of its organization based on single-axon tracing studies. *Proc. Natl. Acad. Sci. U S A.* 102 11888–11893. 10.1073/pnas.0502710102 16087877 PMC1187973

[B69] Lin J. Y. Knutsen P. M. Muller A. Kleinfeld D. Tsien R. Y. (2013). ReaChR: A red-shifted variant of channelrhodopsin enables deep transcranial optogenetic excitation. *Nat. Neurosci.* 16 1499–1508. 10.1038/nn.3502 23995068 PMC3793847

[B70] Liu F. Graybiel A. M. (1992). Heterogeneous development of calbindin-D_28*K*_ expression in the striatal matrix. *J. Comp. Neurol.* 320 304–322. 10.1002/cne.903200304 1351896

[B71] Madisen L. Garner A. R. Shimaoka D. Chuong A. S. Klapoetke N. C. Li L. (2015). Transgenic mice for intersectional targeting of neural sensors and effectors with high specificity and performance. *Neuron* 85 942–958. 10.1016/j.neuron.2015.02.022 25741722 PMC4365051

[B72] Mallet N. Micklem B. R. Henny P. Brown M. T. Williams C. Bolam J. P. (2012). Dichotomous organization of the external globus pallidus. *Neuron* 74 1075–1086. 10.1016/j.neuron.2012.04.027 22726837 PMC3407962

[B73] Mallet N. Schmidt R. Leventhal D. Chen F. Amer N. Boraud T. (2016). Arkypallidal cells send a stop signal to striatum. *Neuron* 89 308–316. 10.1016/j.neuron.2015.12.017 26777273 PMC4871723

[B74] Mastro K. J. Zitelli K. T. Willard A. M. Leblanc K. H. Kravitz A. V. Gittis A. H. (2017). Cell-specific pallidal intervention induces long-lasting motor recovery in dopamine-depleted mice. *Nat. Neurosci.* 20 815–823. 10.1038/nn.4559 28481350 PMC5546121

[B75] Matsuda W. Furuta T. Nakamura K. C. Hioki H. Fujiyama F. Arai R. (2009). Single nigrostriatal dopaminergic neurons form widely spread and highly dense axonal arborizations in the neostriatum. *J. Neurosci.* 29 444–453. 10.1523/JNEUROSCI.4029-08.2009 19144844 PMC6664950

[B76] McGregor M. M. McKinsey G. L. Girasole A. E. Bair-Marshall C. J. Rubenstein J. L. R. Nelson A. B. (2019). Functionally distinct connectivity of developmentally targeted striosome neurons. *Cell Rep.* 29 1419–1428 e5. 10.1016/j.celrep.2019.09.076 31693884 PMC6866662

[B77] Miura M. Masuda M. Aosaki T. (2008). Roles of μ-opioid receptors in GABAergic synaptic transmission in the striosome and matrix compartments of the striatum. *Mol. Neurobiol.* 37 104–115. 10.1007/s12035-008-8023-2 18473190

[B78] Miyamoto Y. Katayama S. Shigematsu N. Nishi A. Fukuda T. (2018). Striosome-based map of the mouse striatum that is conformable to both cortical afferent topography and uneven distributions of dopamine D1 and D2 receptor-expressing cells. *Brain Struct. Funct.* 223 4275–4291. 10.1007/s00429-018-1749-3 30203304 PMC6267261

[B79] Mizutani K. Takahashi S. Okamoto S. Karube F. Fujiyama F. (2017). Substance P effects exclusively on prototypic neurons in mouse globus pallidus. *Brain Struct. Funct.* 222 4089–4110. 10.1007/s00429-017-1453-8 28608288

[B80] Paxinos G. Watson C. (2013). *The rat brain in stereotaxic coordinates*, 7th Edn. Amsterdam: Academic Press.

[B81] Petreanu L. Mao T. Sternson S. M. Svoboda K. (2009). The subcellular organization of neocortical excitatory connections. *Nature* 457 1142–1145. 10.1038/nature07709 19151697 PMC2745650

[B82] Prager E. M. Dorman D. B. Hobel Z. B. Malgady J. M. Blackwell K. T. Plotkin J. L. (2020). Dopamine oppositely modulates state transitions in striosome and matrix direct pathway striatal spiny neurons. *Neuron* 108 1091–1102.e5. 10.1016/j.neuron.2020.09.028 33080228 PMC7769890

[B83] Ragsdale C. W. Graybiel A. M. (1990). A simple ordering of neocortical areas established by the compartmental organization of their striatal projections. *Proc. Natl. Acad. Sci. U S A.* 87 6196–6199. 10.1073/pnas.87.16.6196 1696719 PMC54499

[B84] Ragsdale C. W. Graybiel A. M. (1991). Compartmental organization of the thalamostriatal connection in the cat. *J. Comp. Neurol.* 311 134–167. 10.1002/cne.903110110 1719043

[B85] Raju D. V. Shah D. J. Wright T. M. Hall R. A. Smith Y. (2006). Differential synaptology of vGluT2-containing thalamostriatal afferents between the patch and matrix compartments in rats. *J. Comp. Neurol.* 499 231–243. 10.1002/cne.21099 16977615 PMC2571956

[B86] Ratna D. D. Francis T. C. (2025). Extrinsic and intrinsic control of striatal cholinergic interneuron activity. *Front. Mol. Neurosci.* 18:1528419. 10.3389/fnmol.2025.1528419 40018010 PMC11865219

[B87] Redgrave P. Vautrelle N. Reynolds J. N. (2011). Functional properties of the basal ganglia’s re-entrant loop architecture: Selection and reinforcement. *Neuroscience* 198 138–151. 10.1016/j.neuroscience.2011.07.060 21821101

[B88] Rothman J. S. Silver R. A. (2018). NeuroMatic: An integrated open-source software toolkit for acquisition, analysis and simulation of electrophysiological data. *Front. Neuroinform* 12:14. 10.3389/fninf.2018.00014 29670519 PMC5893720

[B89] Sadikot A. F. Parent A. Smith Y. Bolam J. P. (1992). Efferent connections of the centromedian and parafascicular thalamic nuclei in the squirrel monkey: A light and electron microscopic study of the thalamostriatal projection in relation to striatal heterogeneity. *J. Comp. Neurol.* 320 228–242. 10.1002/cne.903200207 1619051

[B90] Sato F. Lavallee P. Levesque M. Parent A. (2000). Single-axon tracing study of neurons of the external segment of the globus pallidus in primate. *J. Comp. Neurol.* 417 17–31. 10660885

[B91] Schindelin J. Arganda-Carreras I. Frise E. Kaynig V. Longair M. Pietzsch T. (2012). Fiji: an open-source platform for biological-image analysis. *Nat. Methods* 9, 676–682. 10.1038/nmeth.2019 22743772 PMC3855844

[B92] Sciolino N. R. Hsiang M. Mazzone C. M. Wilson L. R. Plummer N. W. Amin J. (2022). Natural locus coeruleus dynamics during feeding. *Sci. Adv.* 8:eabn9134. 10.1126/sciadv.abn9134 35984878 PMC9390985

[B93] Shipp S. (2017). The functional logic of corticostriatal connections. *Brain Struct. Funct.* 222 669–706. 10.1007/s00429-016-1250-9 27412682 PMC5334428

[B94] Shu Y. Yu Y. Yang J. McCormick D. A. (2007). Selective control of cortical axonal spikes by a slowly inactivating K+ current. *Proc. Natl. Acad. Sci. U S A*. 104 11453–11458. 10.1073/pnas.0702041104 17581873 PMC2040919

[B95] Silberberg G. Bolam J. P. (2015). Local and afferent synaptic pathways in the striatal microcircuitry. *Curr. Opin. Neurobiol.* 33 182–187. 10.1016/j.conb.2015.05.002 26051382

[B96] Smith J. B. Klug J. R. Ross D. L. Howard C. D. Hollon N. G. Ko V. I. (2016). Genetic-based dissection unveils the inputs and outputs of striatal patch and matrix compartments. *Neuron* 91 1069–1084. 10.1016/j.neuron.2016.07.046 27568516 PMC5017922

[B97] Smith Y. Parent A. (1986). Differential connections of caudate nucleus and putamen in the squirrel monkey (Saimiri sciureus). *Neuroscience* 18 347–371. 10.1016/0306-4522(86)90159-4 3736862

[B98] Smith Y. Galvan A. Ellender T. J. Doig N. Villalba R. M. Huerta-Ocampo I. (2014). The thalamostriatal system in normal and diseased states. *Front. Syst. Neurosci.* 8:5. 10.3389/fnsys.2014.00005 24523677 PMC3906602

[B99] Spix T. A. Nanivadekar S. Toong N. Kaplow I. M. Isett B. R. Goksen Y. (2021). Population-specific neuromodulation prolongs therapeutic benefits of deep brain stimulation. *Science* 374 201–206. 10.1126/science.abi7852 34618556 PMC11098594

[B100] Suryanarayana S. M. Hellgren Kotaleski J. Grillner S. Gurney K. N. (2019). Roles for globus pallidus externa revealed in a computational model of action selection in the basal ganglia. *Neural Netw.* 109 113–136. 10.1016/j.neunet.2018.10.003 30414556

[B101] Susaki E. A. Shimizu C. Kuno A. Tainaka K. Li X. Nishi K. (2020). Versatile whole-organ/body staining and imaging based on electrolyte-gel properties of biological tissues. *Nat. Commun.* 11:1982. 10.1038/s41467-020-15906-5 32341345 PMC7184626

[B102] Susaki E. A. Tainaka K. Perrin D. Yukinaga H. Kuno A. Ueda H. R. (2015). Advanced CUBIC protocols for whole-brain and whole-body clearing and imaging. *Nat. Protoc.* 10 1709–1727. 10.1038/nprot.2015.085 26448360

[B103] Tepper J. M. Koós T. Ibanez-Sandoval O. Tecuapetla F. Faust T. W. Assous M. (2018). Heterogeneity and diversity of striatal GABAergic interneurons: Update 2018. *Front. Neuroanat.* 12:91. 10.3389/fnana.2018.00091 30467465 PMC6235948

[B104] Tepper J. M. Tecuapetla F. Koos T. Ibanez-Sandoval O. (2010). Heterogeneity and diversity of striatal GABAergic interneurons. *Front. Neuroanat.* 4:150. 10.3389/fnana.2010.00150 21228905 PMC3016690

[B105] Unzai T. Kuramoto E. Kaneko T. Fujiyama F. (2017). Quantitative analyses of the projection of individual neurons from the midline thalamic nuclei to the striosome and matrix compartments of the rat striatum. *Cereb. Cortex* 27 1164–1181. 10.1093/cercor/bhv295 26672610

[B106] Vitek J. L. Zhang J. Hashimoto T. Russo G. S. Baker K. B. (2012). External pallidal stimulation improves parkinsonian motor signs and modulates neuronal activity throughout the basal ganglia thalamic network. *Exp. Neurol.* 233 581–586. 10.1016/j.expneurol.2011.09.031 22001773 PMC3536483

[B107] Walker R. H. Arbuthnott G. W. Baughman R. W. Graybiel A. M. (1993). Dendritic domains of medium spiny neurons in the primate striatum: Relationships to striosomal borders. *J. Comp. Neurol.* 337 614–628. 10.1002/cne.903370407 8288774

[B108] Wilson C. J. (1987). Morphology and synaptic connections of crossed corticostriatal neurons in the rat. *J. Comp. Neurol.* 263 567–580. 10.1002/cne.902630408 2822779

[B109] Yoshida A. Hikosaka O. (2025). Excitatory drive to the globus pallidus external segment facilitates action initiation in non-human primates. *Curr. Biol.* 35 4321–4336.e6. 10.1016/j.cub.2025.07.051 40816282 PMC12360488

[B110] Yoshizawa T. Ito M. Doya K. (2018). Reward-predictive neural activities in striatal striosome compartments. *eNeuro* 5:ENEURO.0367-17.2018. 10.1523/ENEURO.0367-17.2018 29430520 PMC5804148

[B111] Zingg B. Chou X. L. Zhang Z. G. Mesik L. Liang F. Tao H. W. (2017). AAV-mediated anterograde transsynaptic tagging: Mapping corticocollicular input-defined neural pathways for defense behaviors. *Neuron* 93 33–47. 10.1016/j.neuron.2016.11.045 27989459 PMC5538794

[B112] Zingg B. Peng B. Huang J. Tao H. W. Zhang L. I. (2020). Synaptic specificity and application of anterograde transsynaptic AAV for probing neural circuitry. *J. Neurosci.* 40 3250–3267. 10.1523/JNEUROSCI.2158-19.2020 32198185 PMC7159884

